# Adaptations of the 3T3-L1 adipocyte lipidome to defective ether lipid catabolism upon *Agmo* knockdown

**DOI:** 10.1016/j.jlr.2022.100222

**Published:** 2022-05-07

**Authors:** Sabrina Sailer, Katharina Lackner, Mia L. Pras-Raves, Eric J.M. Wever, Jan B. van Klinken, Adriaan D. Dane, Stephan Geley, Jakob Koch, Georg Golderer, Gabriele Werner-Felmayer, Markus A. Keller, Werner Zwerschke, Frédéric M. Vaz, Ernst R. Werner, Katrin Watschinger

**Affiliations:** 1 Institute of Biological Chemistry, Biocenter, Medical University of Innsbruck, Innsbruck, Austria; 2 Laboratory Genetic Metabolic Diseases, Department of Clinical Chemistry and Pediatrics, Emma Children's Hospital, Amsterdam UMC Location University of Amsterdam, Amsterdam, The Netherlands; 3 Core Facility Metabolomics, Amsterdam UMC Location University of Amsterdam, Amsterdam, The Netherlands; 4 Bioinformatics Laboratory, Department of Epidemiology & Data Science, Amsterdam Public Health Research Institute, Amsterdam UMC Location University of Amsterdam, Amsterdam, The Netherlands; 5 Institute of Molecular Pathophysiology, Biocenter, Medical University of Innsbruck, Innsbruck, Austria; 6 Institute of Human Genetics, Medical University of Innsbruck, Innsbruck, Austria; 7 Division of Cell Metabolism and Differentiation Research, Research Institute for Biomedical Aging Research, University of Innsbruck, Innsbruck, Austria; 8 Amsterdam Gastroenterology Endocrinology Metabolism, Inborn Errors of Metabolism, Amsterdam, The Netherlands

**Keywords:** alkylglycerol monooxygenase, ether lipids, 3T3-L1, adipocyte differentiation, lipid metabolism, lipidomics, adipocytes, lipids, triacylglycerol, lipolysis and fatty acid metabolism, Adipoq, adiponectin, Agmo, alkylglycerol monooxygenase, Agps, alkylglycerone phosphate synthase, CE, cholesteryl ester, DEX, dexamethasone, DG, diacylglycerol, Elovl3, elongation of very long chain fatty acids protein 3, Fabp4, fatty acid-binding protein 4, Far1, fatty acyl-CoA reductase 1, Fasn, fatty acidsynthase, Gnpat, glyceronephosphate *O*-acyltransferase, IBMX, 3-isobutyl-1-methylxanthine, Lep, leptin, (L)PC[O]/[P], alkyl-/alkenyl-(lyso)phosphatidylcholine, (L)PE[O]/[P], alkyl-/alkenyl-(lyso)phosphatidylethanolamine, Lpl, lipoprotein lipase, Mgll, monoacylglycerol lipase, PC, phosphatidylcholine, PE, phosphatidylethanolamine, Pnpla2, patatin-like phospholipase domaincontaining 2/adipose triglyceride lipase, Pparg, peroxisome proliferator-activated receptor gamma, RGZ, rosiglitazone, shRNA, short hairpin RNA, TG, triacylglycerol, TG[O/P], alkyl-/alkenyl-diacylglycerol

## Abstract

Little is known about the physiological role of alkylglycerol monooxygenase (AGMO), the only enzyme capable of cleaving the 1-*O*-alkyl ether bond of ether lipids. Expression and enzymatic activity of this enzyme can be detected in a variety of tissues including adipose tissue. This labile lipolytic membrane-bound protein uses tetrahydrobiopterin as a cofactor, and mice with reduced tetrahydrobiopterin levels have alterations in body fat distribution and blood lipid concentrations. In addition, manipulation of AGMO in macrophages led to significant changes in the cellular lipidome, and alkylglycerolipids, the preferred substrates of AGMO, were shown to accumulate in mature adipocytes. Here, we investigated the roles of AGMO in lipid metabolism by studying 3T3-L1 adipogenesis. AGMO activity was induced over 11 days using an adipocyte differentiation protocol. We show that RNA interference-mediated knockdown of AGMO did not interfere with adipocyte differentiation or affect lipid droplet formation. Furthermore, lipidomics revealed that plasmalogen phospholipids were preferentially accumulated upon Agmo knockdown, and a significant shift toward longer and more polyunsaturated acyl side chains of diacylglycerols and triacylglycerols could be detected by mass spectrometry. Our results indicate that alkylglycerol catabolism has an influence not only on ether-linked species but also on the degree of unsaturation in the massive amounts of triacylglycerols formed during in vitro 3T3-L1 adipocyte differentiation.

Ether-linked lipid species such as plasmanyl-glycerophospholipids (alkyl-linked lipids) or plasmenyl-glycerophospholipids (alkenyl-linked lipids or plasmalogens) are known to be important integral membrane constituents in several organs including the brain (1). They carry an exceptionally high amount of polyunsaturated fatty acids at their *sn*-2 position (2), are essential for proper eye development, and play an important role in male fertility (1). Ether lipids interfere with kinase signaling pathways such as protein kinase C (3, 4, 5) or protein kinase B (6). Also platelet-activating factor, an important lipid biomediator, belongs to this lipid class (7, 8). An important subcellular hub for ether lipid synthesis are peroxisomes, which also play a pivotal role in other fatty acid processes including very long-chain and branched-chain fatty acid degradation (9).

The only known enzyme able to degrade plasmanyl ether species is alkylglycerol monooxygenase (AGMO) (10), a highly hydrophobic integral membrane protein (11). Because AGMO enzymatic activity is quickly lost during standard biochemical procedures, protein purification attempts have not succeeded so far (12, 13, 14). In 2010, we were able to identify *Tmem195* as the *Agmo* gene (15), which now enables us to examine its physiological role in detail. AGMO is differentially regulated in mouse macrophage polarization (16, 17), and activity manipulation in a murine macrophage cell line impacts on the composition of the cellular lipidome (17). In the same study, modulations of AGMO activity in murine macrophages led to substantial accumulation of ether-linked phospholipids (plasmanyl and plasmenyl) and alkylglycerols. In earlier analyses, AGMO was also suggested to play a role in platelet-activating factor degradation (16). In the model organism *Caenorhabditis elegans*, mutants deficient for AGMO showed a more fragile cuticle and an altered sensitivity to bacterial infection (18). Analysis of the cuticle lipid profile revealed alterations of ester lipids, glucosylceramides, a lower abundance of negatively charged lipid headgroups, and accumulation of higher molecular weight lipids with longer side chains (19). Furthermore, it was recently shown that ether-linked phosphatidylcholines and sphingolipids exert an inverse function in bidirectional endoplasmic reticulum trafficking of glycosylphosphatidylinositol anchors (20).

Still, the exact physiological role of AGMO is not well understood. From genome-wide association studies in humans and from experimental evidence in model organisms, associations between the *AGMO* locus, and biologically relevant traits like energy homeostasis and infections were found (21). Single nucleotide polymorphisms adjacent to or in the human *AGMO* gene were correlated with fasting glucose levels (22) and with recurrent leishmaniasis (23), respectively. Manipulation of tetrahydrobiopterin levels, a crucial redox partner of AGMO, in mouse models showed that a complete cofactor deficiency leads to embryonic lethality (24). If, however, modest tetrahydrobiopterin levels are maintained in mice during pregnancy, pups are born normally but have more body fat and altered fat distribution, as well as elevated blood glucose and cholesterol levels (25). Recently, we succeeded in generation of the first *Agmo* knockout mouse model to study the physiological relevance of ether lipid degradation by AGMO in more detail (26).

*Agmo* is abundantly expressed and active in many tissues of rats and mice, including liver, gastrointestinal tract, and different fat tissues (15, 27). There are reports on ether lipids in adipocytes and adipogenesis claiming that incorporation of ether-linked lipid species, such as ethanolamine plasmalogens, helps adipocytes to maintain their membrane rigidity (28). Another clue that ether lipids are relevant for adipose tissue came from lipidomic analyses of human plasma and adipose tissue samples, which revealed that levels of alkyl-linked and alkenyl-linked phospholipids are changed in obese compared with lean individuals (29, 30, 31). As compared with ether-linked phospholipids, the general role of neutral ether lipids like 1-*O*-alkyl-2,3-diacylglycerols (DGs)—the ether analogues of triacylglycerols (TGs)—in physiology, however, is only marginally understood. These neutral ether lipids were shown to be upregulated in vitro in cell models of adipocyte differentiation (32), to exert a proadipogenic stimulus in 3T3-L1 adipogenesis (33) and to be able to rescue peroxin 16 deficiency-mediated inhibition of adipocyte development (34, 35). In adipocytes, peroxisomes, the crucial organelles for the initial steps of ether lipid biosynthesis, and lipid droplets get in close proximity (36) and are essential for bidirectional lipid trafficking of ether-linked triradylglycerols (TG, DG[O], and DG[P]) to lipid droplets (37).

In light of these few scattered reports about the proadipogenic effect of alkylglycerols, as well as the putative connection of AGMO and obesity including its comorbidities, we studied *Agmo* expression and activity in 3T3-L1 adipocyte differentiation and knocked down its expression by RNA interference in 3T3-L1 preadipocytes, monitored consequences on differentiation, and found quite unexpected impacts of decreased AGMO activity on the global cellular lipidome.

## Materials and methods

### Cell lines and cell culture

The 3T3-L1 preadipocyte cell line (American Type Culture Collection, Manassas, VA) was grown in sterile 75 cm^2^ polystyrene, cell+ growth surface flasks equipped with ventilated screw caps (Sarstedt, Nümbrecht, Germany) in basal medium (DMEM/GlutaMAX high glucose plus sodium pyruvate [Fisher Scientific, Vienna, Austria]) supplemented with 10% fetal bovine serum (Fisher Scientific) and 1% penicillin/streptomycin (Sigma, Vienna, Austria). Cells were split when reaching about 90% confluence with 1× trypsin-EDTA solution (Sigma) and were transferred to collagen-coated 6-well or 96-well plates, which were coated with collagen type I (rat tail; Fisher Scientific, 80 μg/ml working solution in 20 mM acetic acid) overnight at 4°C and afterward washed once with 1× PBS.

For adipocyte differentiation, 5-day postconfluent 3T3-L1 cells were exposed for 3 days to the differentiation medium 1 consisting of basal medium supplemented with 34.4 nM insulin (Sigma), 0.25 μM dexamethasone (DEX) (Sigma), 0.5 mM 3-isobutyl-1-methylxanthine (IBMX) (Sigma), and 2 μM rosiglitazone (RGZ) (Cayman, Tallinn, Estonia). On day 4, the medium was changed to differentiation medium 2 (basal medium supplemented with 34.4 nM insulin only) for the rest of the differentiation protocol until day 11.

### Lipid droplet staining of mature adipocytes

To quantify the amount of lipid droplets and cell nuclei, 3T3-L1 adipocytes were stained with Bodipy™ 493/503 (Fisher Scientific, Invitrogen^TM^, and Molecular Probes^TM^) and Hoechst 33342 (Sigma). For this, cells were washed once with 1× PBS and fixed for 10 min in 4% paraformaldehyde (Merck, Darmstadt, Germany). After fixation, cells were washed twice with 1× PBS and then incubated for 15 min in the dark with a staining solution consisting of 2 μM Bodipy, 2 μg/ml Hoechst, and 1× PBS. Thereafter, cells were again washed twice and then stored in 1× PBS. Images were recorded on a Leica DM IL LED inverted fluorescence microscope (Leica, Wetzlar, Germany). All images were evaluated using the CellProfiler™ cell image analysis software (38). Alternatively, lipid droplets of 3T3-L1 adipocytes were stained with the neutral lipid dye Oil Red O (Sigma). For this, fixed cells were washed twice with 1× PBS and once with 60% triethylphosphate (Sigma) solution in aqua destillata. A 0.5% Oil Red O solution was prepared in 60% triethylphosphate solution and added to the cells for 10 min. Afterward, the Oil Red O staining solution was aspirated and 1× PBS was added for 2 min and changed for fresh 1× PBS.

### RNA isolation and quantitative PCR

Total RNA from 3T3-L1 cells was prepared using the RNeasy Plus Mini Kit according to the manufacturer’s protocol (Qiagen, Hilden, Germany). Transcription into complementary DNA was performed using the M-MLV reverse transcriptase (RNase H Minus, Point Mutant; Promega, Mannheim, Germany) and random hexamer primers (Microsynth, Balgach, Switzerland). For quantitative PCR (qPCR), the TaqMan assay technology using Brilliant III Ultra-Fast QPCR Master Mix (Agilent Technologies, Vienna, Austria) and the Mx3005P qPCR system (Agilent) were used. TaqMan probes were labeled with fluorescein (FAM) (5′) and tetramethylrhodamine (TAMRA) (3′). Primer and TaqMan probe sequences are listed in supplemental Table S1.

### Western blot

Cell pellets were collected in 0.1 M Tris/0.25 M sucrose at day 0 and day 11 of adipocyte differentiation and assessed for protein content by Bradford assay using BSA as standard. The rest of the sample was mixed with 5× SDS sample buffer, and homogenates were sonicated and boiled for 5 min at 95°C. Twenty micrograms of samples were separated on a Novex™ WedgeWell™ 4–20% Tris-Glycine Gel (Fisher Scientific, Invitrogen), blotted onto PVDF membrane (Bio-Rad Laboratories, Inc, Hercules, CA), blocked with 5% skim milk (Sigma), and stained with either mouse anti-fatty acid binding protein 4 (FABP4) (1:1,000 dilution; Santa Cruz, Heidelberg, Germany) or mouse anti-PPARγ (1:500 dilution; Santa Cruz). For the loading control β-actin, mouse anti-actin (1:2,500 dilution; Millipore) was used. As secondary antibody, HRP-linked anti-mouse IgG (Promega) was applied. Blots were incubated with Westar Supernova ECL reagent (LabConsulting, Cyanagen, Bologna, Italy), and signals were recorded with the MicroChemi 4.2 chemiluminescent station (DNR, Neve Yamin, Israel). Western blot band pixel count was quantified with ImageQuant TL software (GE Healthcare Life Sciences, Vienna, Austria), and signals were normalized to the β-actin reference.

### AGMO activity assay

Enzyme activity was measured as described in a previous work (27) with some modifications: Homogenates of 3T3-L1 cells were not centrifuged, and a protein concentration of >1 mg/ml was used to measure the enzymatic activity. These optimization steps were necessary to minimize quenching of accumulated lipids during adipocyte differentiation and to robustly detect the AGMO activity of in vitro differentiated adipocytes. Furthermore, fatty aldehyde dehydrogenase, essential for full conversion of the fatty aldehyde to the fatty acid, was added in its recombinant form to the assay mixture (39). We carefully analyzed samples and controls of each replicate in parallel to exclude artifacts by day-to-day variability of the assay.

### Manipulation of AGMO activity by lentiviral constructs

Stable knockdown of *Agmo* gene expression using shRNAs was performed as already described in a previous work (17). In brief, the pHR-SFFV-DEST-ires-Puro transfer plasmid containing the shRNA-encoding oligonucleotides of murine *Agmo* 1699–1717 for sh*Agmo*1699 (GeneBank accession no.: NM_178767.5) was added to human embryonic kidney 293T cells together with the packaging plasmid (pSPAX2) and the pseudotyping vector pVSV-G for generation of infectious lentiviral particles. The infectious supernatant was harvested at 48 and 72 h after transfection, 0.45 μm filtered, and added to the target cells for 24 h. The sh*Luc* control cell line expressing shRNA against luciferase (155–173 from pGL3 Luciferase; Promega) was generated in parallel. Afterward, cells were selected for puromycin resistance (3.5 μg/ml).

### Cellular lipidomics analysis

For lipidomics analysis, sh*Luc* and sh*Agmo* cells were harvested at day 0 and 11 of adipocyte differentiation. Cells were first washed once with PBS containing 0.5% fatty acid-free BSA (Sigma) and afterward washed with PBS only and trypsinized. Dry cell pellets were snap-frozen in liquid nitrogen and shipped to Amsterdam UMC (the Netherlands) for lipidomic analysis performed in the Core Facility Metabolomics and processing done with an in-house developed pipeline written in R (40, 41, 42). Internal standards for (phospho)lipid classes were added at known concentrations to each sample allowing identification and normalization of intensities (43). Other lipid classes, for which no internal standard was available, were also annotated but excluded from the primary analysis. As quenching effects can differently affect the internal standard versus the analyzed lipids of the associated lipid class, comparisons between unrelated lipid classes should be made with caution. Relative abundances of the same lipid classes were calculated according to the assumptions that the response was similar as compared with their respective internal standard. Therefore, comparisons of relative concentrations between different species are not recommended. Only the same species between different sample groups should be compared (e.g., sh*Luc* vs. sh*Agmo*).

### Statistical analysis

Unless indicated otherwise, data are presented as mean ± SEM. Boxplots show the median ± interquartile range with the whiskers spreading from minimum to maximum. Gaussian distributed data were compared by Student’s *t*-test or by two-way ANOVA without correction for multiple comparisons. For nonparametric data, Kruskal-Wallis test for multiple comparisons or Kolmogorov-Smirnov test for *t*-test was applied. GraphPad Prism 5.01 (GraphPad Software, Inc, San Diego, CA) or Microsoft Excel 2010 (Microsoft Corporation, Redmond, WA) was used. *P* values <0.05 were considered as statistically significant. ∗*P* < 0.05, ∗∗*P* < 0.01, ∗∗∗*P* < 0.001, and ∗∗∗∗*P* < 0.0001.

## Results

### AGMO is induced in 3T3-L1 adipocyte differentiation

**Fig. 1:**
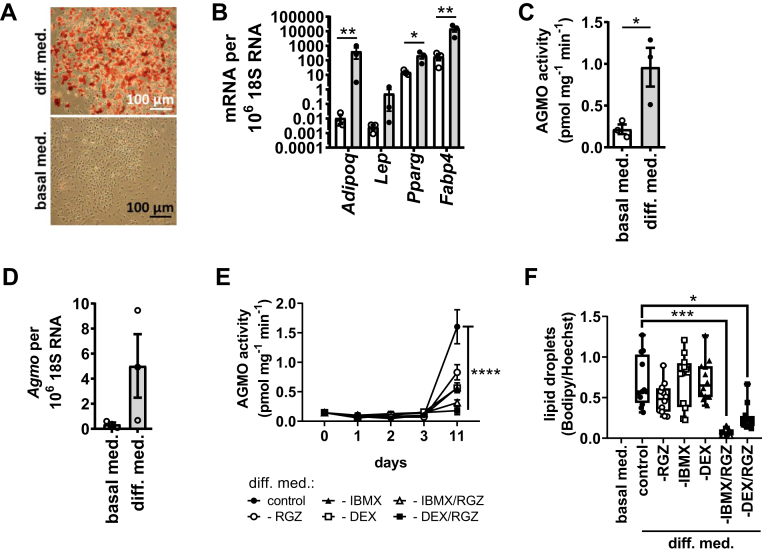
Determination of adipocyte-specific markers and AGMO activity during 3T3-L1 adipocyte differentiation. A: Oil Red O staining of differentiated (day 11, upper panel) and undifferentiated (day 0, lower panel) 3T3-L1 cells. B: Gene expression of late adipocyte markers adiponectin (*Adipoq*), leptin (*Lep*), peroxisome proliferator-activated receptor gamma (*Pparg*), and fatty acid-binding protein 4 (*Fabp4*) was analyzed by RT-qPCR using TaqMan technology. Open bars and open circles represent undifferentiated cells at day 0 prior to differentiation; gray bars, full circles correspond to mature adipocytes at day 11 after exposure of 3T3-L1 cells to the adipocyte differentiation medium 1 and 2 (n = 3). C: AGMO enzymatic activity in cell pellets of 3T3-L1 exposed to the basal medium at day 0 (open bars and open circles) and cells supplemented with the differentiation medium 1 and 2 at day 11 (gray bars and full circles) of adipogenesis (n = 3). D: *Agmo* gene expression of cells at day 0 (open bars and open circles) and day 11 (gray bars and full circles) of adipocyte differentiation. E: Time course of AGMO activity during differentiation of 3T3-L1 cells exposed to the standard hormonal differentiation cocktail (diff. med. control) or a hormonal induction medium devoid of either IBMX, RGZ, DEX, or IBMX/RGZ and DEX/RGZ in combination (n = 5). F: Lipid droplet quantification by Bodipy and Hoechst staining using the CellProfiler™ analysis software of 3T3-L1 cells at day 0 and day 11 incubated with the complete differentiation medium (control) or differentiation medium with omitted IBMX, RGZ, DEX, or IBMX/RGZ and DEX/RGZ (three areas per well; n = 4). Mean ± SEM except for the boxplots in F, which show the median ± interquartile range (IQR). Whiskers range from minimum to maximum. ∗*P* < 0.05, ∗∗*P* < 0.01, ∗∗∗*P* < 0.001, and ∗∗∗∗*P* < 0.0001.

We assessed *Agmo* expression and enzymatic activity in 3T3-L1 cells kept either in basal medium (day 0) or in differentiation medium (day 11) supplemented with RGZ, IBMX, DEX as well as insulin for the first 3 days and from day 4 supplemented with insulin only. Cells were harvested at days 0 and 11. 3T3-L1 preadipocytes differentiated robustly as monitored by Oil Red O staining (Fig. 1A). Successful adipocyte differentiation, validated by increased expression of four late adipocyte markers (peroxisome proliferator-activated receptor gamma [*Pparg*]: 14-fold, *P* = 0.0135; adiponectin [*Adipoq*]: 38,000-fold, *P* = 0.0057; fatty acid-binding protein 4 [*Fabp4*]: 82-fold, *P* = 0.0076; leptin [*Lep*]: 192-fold, *P* = 0.0724; compared with cells at day 0, Fig. 1B), also induced AGMO expression and enzymatic activity (enzyme activity: 4-fold, *P* = 0.036; gene expression: 14-fold, *P* = 0.0614; Fig. 1C, D). To examine whether AGMO activity was selectively induced by any of the hormones contained in the differentiation medium 1, components were omitted from the differentiation cocktail and cells were collected at day 0, 1, 2, 3, and 11 of adipocyte differentiation and analyzed for enzyme activity. We observed that omission of a single hormone already led to significantly decreased AGMO activities (control: 1.60 ± 0.29 pmol mg^−1^ min^−1^; −RGZ: 0.83 ± 0.13 pmol mg^−1^ min^−1^; −IBMX: 0.57 ± 0.08 pmol mg^−1^ min^−1^; −DEX: 0.56 ± 0.03 pmol mg^−1^ min^−1^, *P* < 0.0001, Fig. 1E), whereas lipid droplet formation at day 11 was similar to the control (Fig. 1F). However, when combinations of either IBMX and RGZ or DEX and RGZ were omitted, AGMO activities were not induced anymore and remained at values comparable to those measured at day 0 (−IBMX/RGZ: 0.31 ± 0.05 pmol mg^−1^ min^−1^; −DEX/RGZ: 0.18 ± 0.07 pmol mg^−1^ min^−1^, *P* < 0.0001; control day 0: 0.14 ± 0.05 pmol mg^−1^ min^−1^, Fig. 1E). And under these conditions, also lipid droplet formation at day 11 was now severely hampered (−IBMX/RGZ: 89% reduction compared with cells exposed to the complete differentiation medium, *P* = 0.0001; −DEX/RGZ: 63% reduction compared with cells exposed to the complete differentiation medium, *P* = 0.04, Fig. 1F).

### AGMO is not required for cellular adipocyte differentiation

**Fig. 2:**
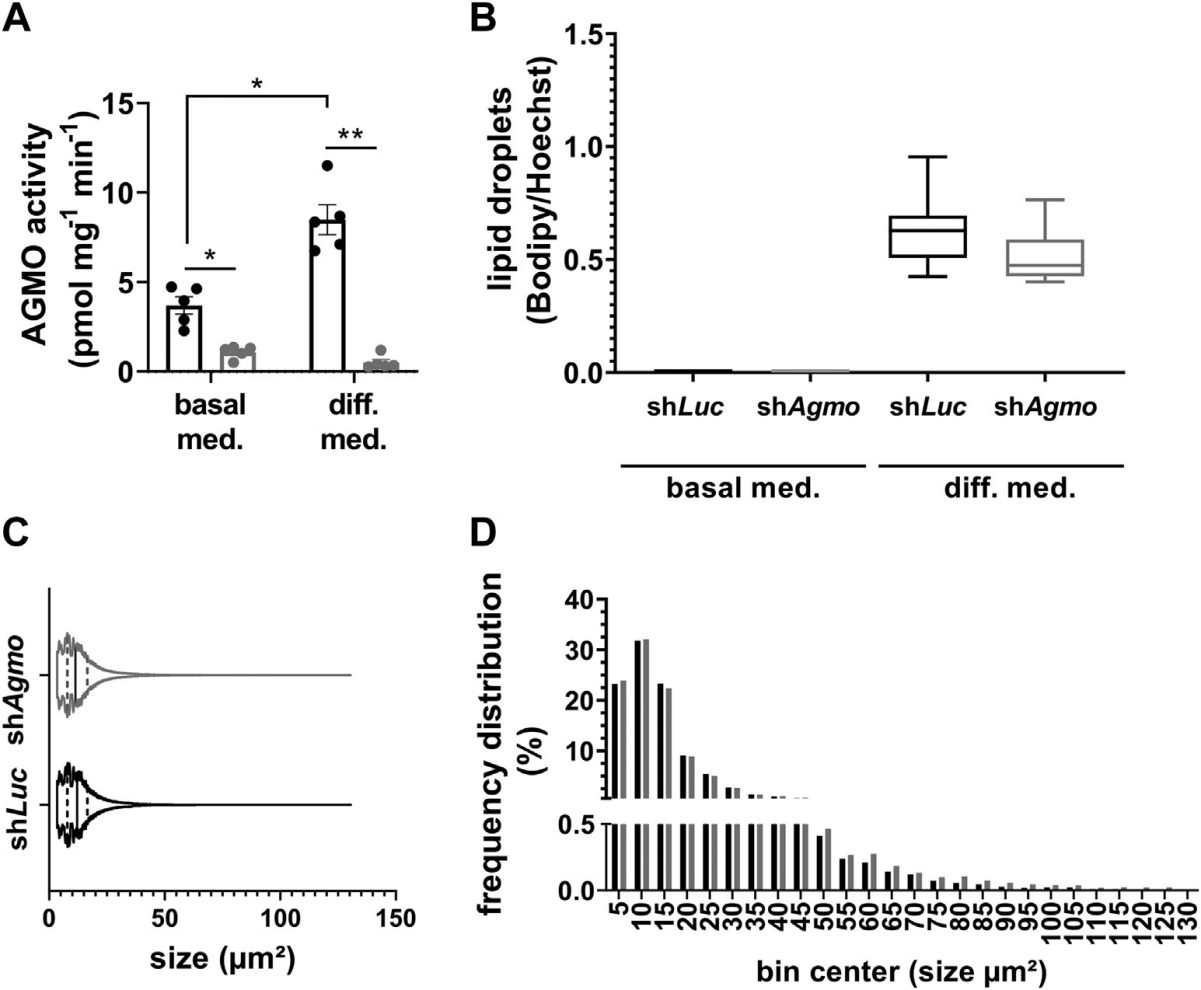
*Agmo* knockdown and the impact on lipid droplet mass and size during adipogenesis. A: AGMO enzymatic activity of 3T3-L1 cells harboring either a knockdown against the firefly luciferase as a control (black bars) or against *Agmo* (gray bars) at day 0 prior to differentiation and day 11 at the end of differentiation when mature adipocytes have formed (n = 5). B: Lipid droplet formation per cell of undifferentiated cells at day 0 and differentiated cells at day 11 using Bodipy for lipid droplet staining and Hoechst to stain cellular nuclei (four areas per well, n = 5). C: Violin plots showing lipid droplet size frequency distribution in the differentiated sh*Luc* cell line (black) and the differentiated sh*Agmo* knockdown cell line (gray) (12 areas per well, two wells per replicate, n = 3). The solid line represents the median and the staggered line the quartiles. D: Histogram showing the relative distribution of the lipid droplet size of sh*Luc* adipocytes and sh*Agmo* adipocytes (n = 3). Black bars and plots are representative for the sh*Luc* control, while sh*Agmo* cell lines are presented in gray. Mean ± SEM except for the boxplots in B, which show the median ± interquartile range (IQR). Whiskers range from minimum to maximum. ∗*P* < 0.05 and ∗∗*P* < 0.01.

Having established that AGMO expression and activity is induced during 3T3-L1 adipocyte differentiation, we knocked down AGMO in these cells by infecting them with a lentiviral construct, which targets position 1,699–1,717 (sh*Agmo*) of the murine *Agmo* mRNA (17). In parallel, we generated a control line with a shRNA targeted against the firefly luciferase mRNA (sh*Luc*). We exposed both the sh*Agmo* and sh*Luc* cell lines to the standard differentiation cocktail and quantified AGMO activity (Fig. 2A), lipid droplet formation at day 0 and day 11 (Fig. 2B), and lipid droplet size (Fig. 2C, D). AGMO enzymatic activities were significantly reduced in the knockdown cell line compared with the sh*Luc* control line in both the undifferentiated and differentiated state (day 0: 3-fold decrease, *P* = 0.011; day 11: 17-fold decrease, *P* = 0.005). Despite the decreased enzyme activity, *Agmo* knockdown did not influence adipocyte differentiation as shown by quantification of lipid droplet mass and size (Fig. 2B–D). We also looked at gene expression of late adipocyte markers (*Pparg* and *Fabp4*) as well as lipogenic and lipolytic enzymes involved in ester lipid (*Elovl3* [elongation of very long-chain fatty acid protein 3], *Fasn* [fatty acid synthase], *Lpl* [lipoprotein lipase], *Pnpla2* [patatin-like phospholipase domain containing 2/adipose triglyceride lipase], and *Mgll* [monoacylglycerol lipase]) and ether lipid (*Gnpat* [glyceronephosphate *O*-acyltransferase], *Agps* [alkylglycerone phosphate synthase], and *Far1* [fatty acyl-CoA reductase 1]) metabolism (supplemental Fig. S1). Like lipid droplet formation, *Agmo* knockdown did not influence expression of these 10 selected genes at day 0 and day 11 of the differentiation protocol. We also confirmed the significant upregulation of adipocyte markers PPARγ (isoform 1 and 2) and FABP4 on protein expression by Western blot analysis (supplemental Fig. S2).

### *Agmo* knockdown leads to structural rearrangements of neutral lipids

**Fig. 3:**
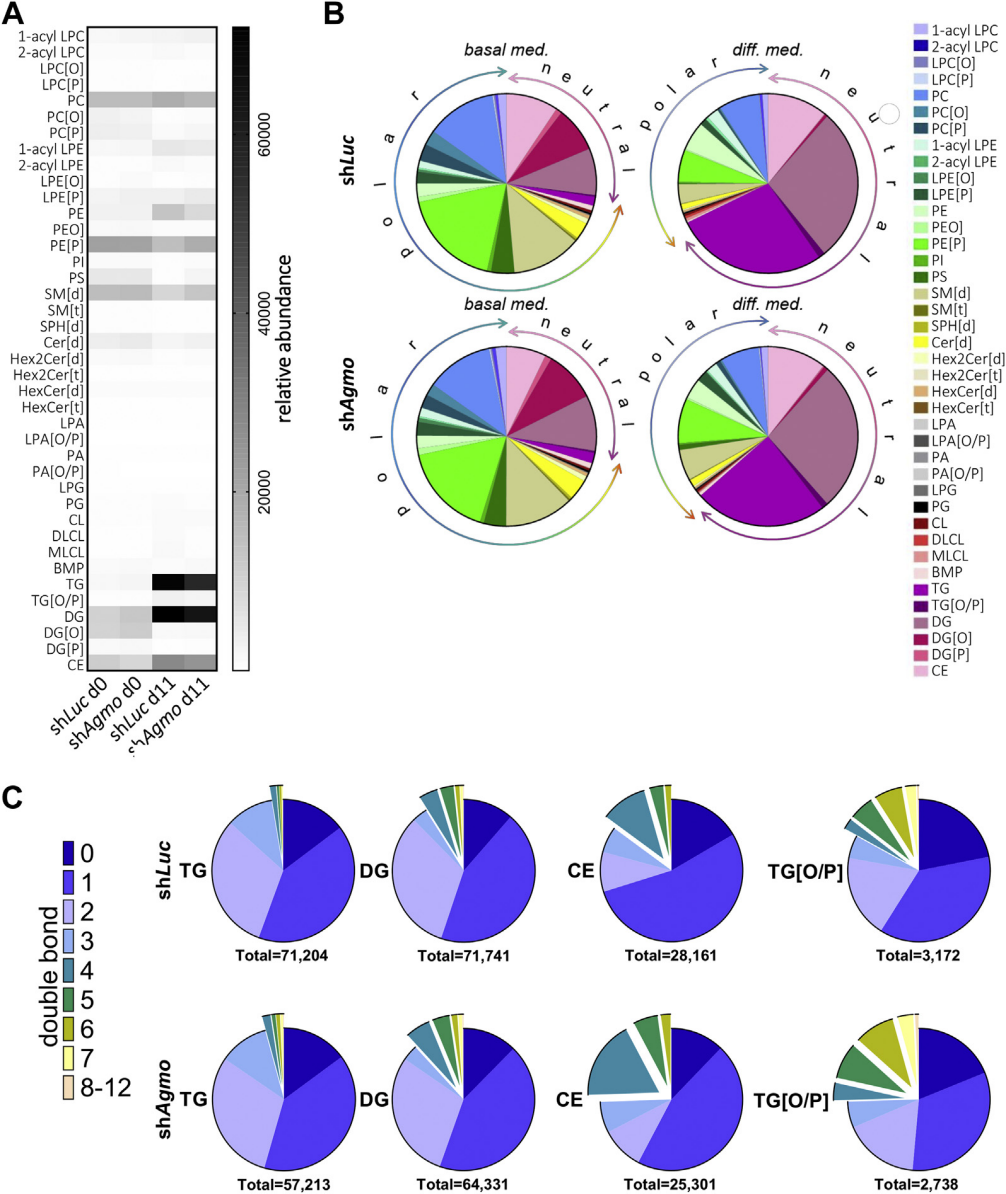
Remodeling of the cellular lipidome during adipocyte differentiation. A: Heatmap representation of the relative abundances of the 40 analyzed lipid classes for control (sh*Luc*) and *Agmo* knockdown (sh*Agmo*) after 0 and 11 days (d0 and d11). B: Pie chart of the relative abundances of the 40 major lipid classes during differentiation of the sh*Luc* control and sh*Agmo* from day 0 to day 11. All species are colored according to their respective lipid class indicated in the color legend on the right. C: Pie charts showing the relative abundance of neutral lipid species sorted according to their degree of desaturation in sh*Luc* and sh*Agmo* in vitro differentiated adipocytes at day 11. Total values below each chart indicate the cumulative relative abundance of all identified single lipid species that are shared in both cell lines and form the respective lipid class. Mean of N = 5 is shown.

**Fig. 4:**
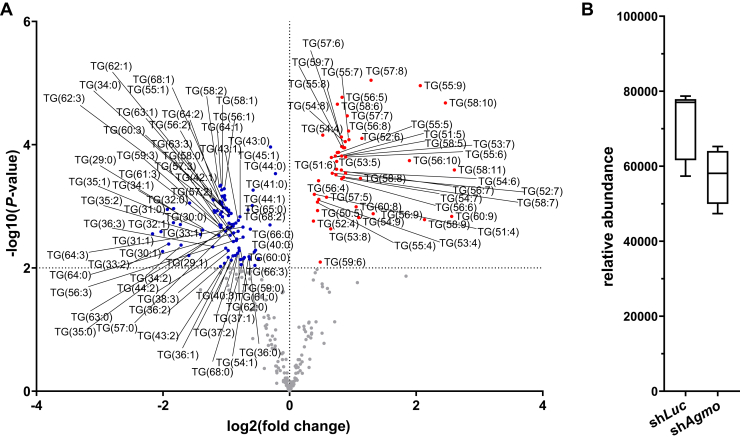
Detailed analysis of carbon chain length and saturation of single TG species upon *Agmo* knockdown in mature adipocytes. A: Volcano plot depicting the lipidomics data of TGs at day 11 of adipocyte differentiation. Significance cutoff is *P* < 0.01 as indicated by the horizontal dotted line. The vertical dotted line separates the log2(fold change) of decreased (blue dots) and increased (red dots) lipid species. B: Boxplots showing the relative abundances of TG at day 11 of adipocyte differentiation in the sh*Luc* and sh*Agmo* cell lines. A full list of accumulated or depleted metabolites is shown in supplemental Table S2.

To understand the role of AGMO in lipid metabolism during adipocyte differentiation, we analyzed the behavior of individual lipid species during adipocyte differentiation in sh*Luc* and sh*Agmo* 3T3-L1 by harvesting samples at day 0 and 11 of differentiation and subjecting them to high-resolution liquid chromatographic mass spectrometric analysis. We could identify and quantify 3,763 lipids, of which 2,145 were used for primary analysis. Partial least square discriminant analysis of lipidomics data showed no discrimination of both cell lines sh*Luc* and sh*Agmo* at day 0; however, we observed a clear separation on basis of the sh*Luc* and sh*Agmo* lipidomes at day 11 (supplemental Fig. S3A). Differentiation to adipocytes had an impact on almost 50% of all quantified lipid classes (Fig. 3A, more details are found in the supplemental Fig. S3A, B) with the most obvious changes in the massive accumulation of neutral lipid classes especially TGs, DGs, and cholesteryl esters (CEs) in both cell lines (Fig. 3B). A full list with all lipid classes and their abundances is shown in supplemental data 1. Since partial least square discriminant analysis was able to discriminate between the sh*Luc* and sh*Agmo* adipocyte lipidomes, we analyzed the molecular architecture of lipid subclasses by quantifying the double bond distribution in both sh*Luc* and sh*Agmo* cell lines and uncovered preferential accumulation of more unsaturated lipid species in the neutral lipid classes TG, DG, CE, and TG[O/P] at day 11 in the sh*Agmo* cell line (Fig. 3C and supplemental Table S2). This effect was especially prominent for TG, which contributed approximately one quarter to the overall cellular lipid pool in mature adipocytes (Fig. 3B). Figure 4A shows a volcano plot of the alteration of individual TG species upon *Agmo* knockdown. The sum of TG species, however, was almost not altered (Fig. 4B). For sh*Agmo* adipocytes, we could quantify 300 individual TG species of which 138 were markedly different (*P* < 0.01, see supplemental Table S3 for relative abundances of individual metabolites). We observed that substantially higher numbers of species containing longer fatty acid substituents starting from at least 50 and ranging up to 60 carbon atoms (summed amount of carbon atoms of acyl side chains) and carrying 4–10 double bonds were 2-fold to 10-fold higher in sh*Agmo* adipocytes compared with differentiated sh*Luc* control cells that tended to store more saturated species (on average <58 carbon atoms in the side-chain fatty acids having 0–3 double bonds). On the other hand, species with shorter fatty acid side chains (i.e., 39 of 116 species with less than 50 carbon atoms in sum) with either 0–2 double bonds were considerably reduced upon *Agmo* knockdown (*P* < 0.01). For alkyl-DGs/alkenyl-DGs (TG[O/P]), we detected 167 lipid species, of which 79 were similarly regulated as the TG upon *Agmo* knockdown (cutoff *P* < 0.01, supplemental Fig. S4 and supplemental Table S4). Here as well, longer-chained species with 4–10 double bonds (55 of 125 species with ≥52 carbon atoms) were enriched upon *Agmo* knockdown. Similarly, we identified 31 of 121 metabolites within the DG class that were differentially accumulated or depleted in adipocytes harboring *Agmo* knockdown compared with control adipocytes. Again, we found that the vast majority of species with shorter fatty acid side chains (most species with ≤34 carbon atoms in sum), which carried mostly 0–2 double bonds, were almost 2-fold lower in sh*Agmo* cells compared with sh*Luc* cells. In contrast, longer-chained species (comprising ≥40 carbon atoms of summed radyl side chains) with 4–8 double bonds were 2-fold accumulated in sh*Agmo* cells compared with the sh*Luc* control (supplemental Fig. S5 and supplemental Table S5). The impact of *Agmo* knockdown on ether-linked alkylacylglycerols (DG[O]) was not as pronounced as in the alkyl-DGs/alkenyl-DGs (TG[O/P]) and mostly manifested in accumulation of 34–42 carbon atom species with 4–6 double bonds on average (31 of 61 species of that size). In addition, we also found a preferential incorporation of multiple double bonds (3, 4, 5, 6) in CE species (in total 8 of 56 identified species with side chains consisting of 16–24 carbon atoms) in the *Agmo* knockdown line, whereas other species with 0–2 double bonds were markedly decreased compared with the sh*Luc* control line at day 11 of differentiation (23 individual species in total out of 56 identified metabolites, supplemental Fig. S6 and supplemental Table S6).

**Fig. 5:**
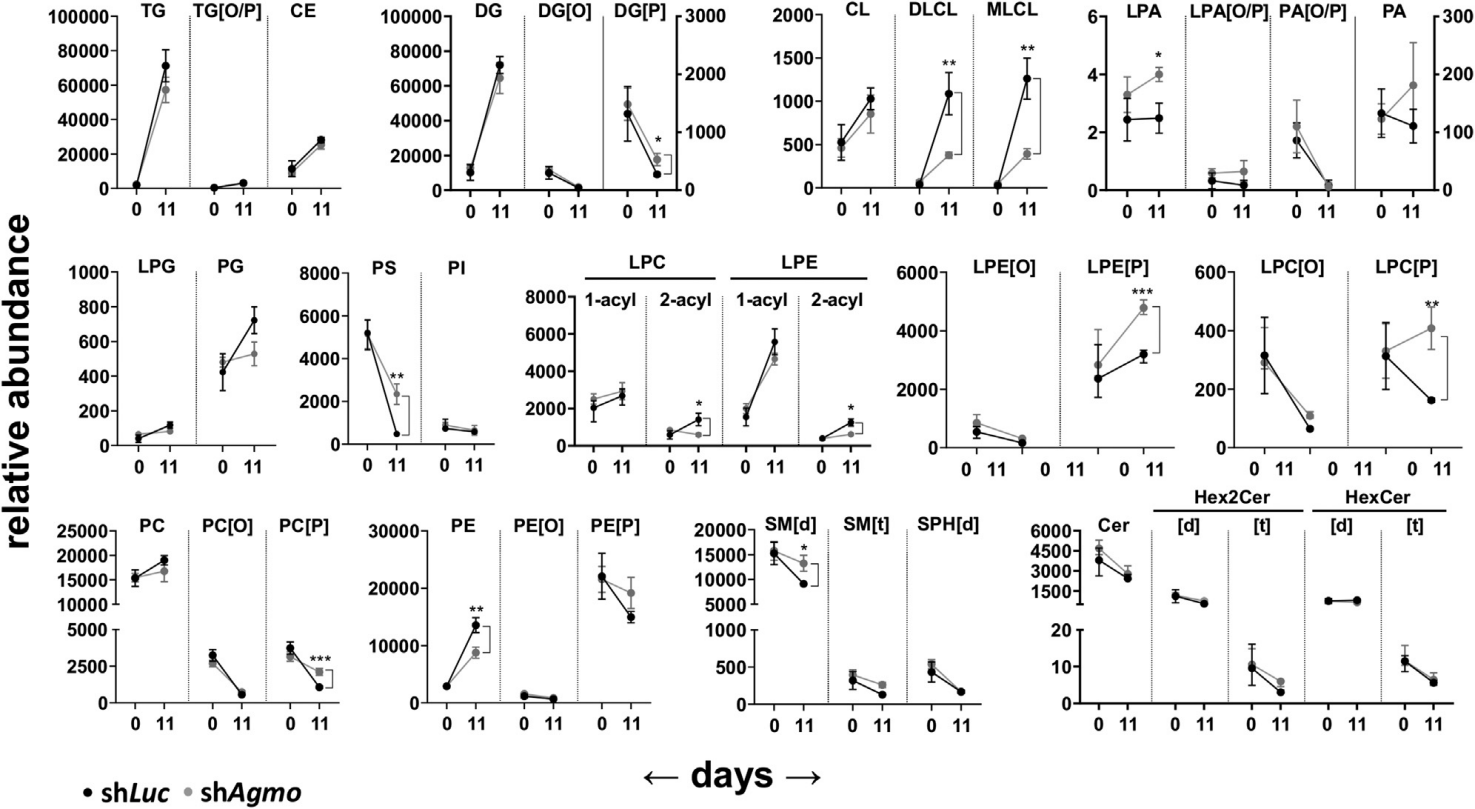
Behavior of lipid classes during 3T3-L1 adipocyte differentiation from day 0 to day 11 of adipocyte differentiation. Black symbols represent the sh*Luc* cell line, and gray symbols depict the sh*Agmo* cell line. Data are presented as mean ± SD, n = 5. ∗*P* < 0.05, ∗∗*P* < 0.01, and ∗∗∗*P* < 0.001.

Figure 5 shows the influence of *Agmo* knockdown on the behavior of all lipid classes during adipocyte differentiation. We observed no substantial changes in relative abundances of potential AGMO substrates between both cell lines (i.e., polar lysophospholipids carrying an alkyl bond like lyso-phosphatidylethanolamine (LPE[O]) or lyso-phosphatidylcholine (LPC[O]) before and after differentiation to adipocytes (Fig. 3A, B and supplemental Fig. S3B). Another subclass that serves AGMO as substrates are the monoalkylglycerols (MG[O]), which however, were not included in the primary dataset because of the lack of available internal standard. For them, we found preferential accumulation of a few selected species carrying intermediate monounsaturated and longer saturated fatty alcohol side chains at *sn*-1 (18:1, 19:1, and 22:0) pointing toward a favored degradation by AGMO for these species (supplemental Fig. S7A, B). Therefore, we also examined the composition of the *sn*-1 attached fatty alcohols in lyso-PC[O] and lyso-PE[O] and found, similarly to the monoalkylglycerol (MG[O]) subclass, favored accumulation of 18:1 side chains in sh*Agmo* adipocytes (supplemental Fig. S8A). In addition, also 16:0 and 18:0 species were enriched and contributed to a substantial amount of the total lipid pool in these classes (supplemental Fig. S8B, C).

While the radyl composition of TG, DG, and CE was altered in *Agmo* knockdown cells, other classes of lipids were affected in their total amount by *Agmo* knockdown. The almost complete disappearance of phosphatidylserine in the sh*Luc* control cells at day 11 was strongly attenuated by *Agmo* knockdown. A similar behavior was also observed for bis(monoacylglycero)phosphate, which was decreased during the course of adipocyte differentiation in the sh*Luc* control cells but stayed at basal levels in sh*Agmo* adipocytes at day 11. 1-*O*-Alkenyl phospholipid species (PC[P] and PE[P]) were increased on day 11 in adipocytes with compromised AGMO activity, with concurrent decreases in the respective ester analogues (PC and PE), whereas the alkyl species (PC[O] and PE[O]) remained unchanged by *Agmo* knockdown.

## Discussion

Here, we show for the first time that AGMO expression as well as activity is induced during 3T3-L1 adipocyte differentiation (Fig. 1). This upregulation of a lipid-cleaving enzyme in parallel to the massive accumulation of its substrates might seem contradictory at first but was also observed for the lipolytic enzymes of the ester lipid degradation pathway, that is, adipocyte triglyceride lipase and monoacylglycerol lipase (44, 45) and most likely points to the necessity of lipid homeostasis and remodeling during and beyond adipocyte differentiation. Yet, the consequences of defective ether lipid degradation during adipocyte differentiation have not been studied so far. We found that knockdown of AGMO, a catabolic enzyme involved in plasmanyl ether lipid metabolism, in 3T3-L1 cells and subsequent differentiation to adipocytes had no major impact on lipid class abundances but strongly impacted on the molecular architecture of many lipid classes beyond those of ether lipids. Cells where *Agmo* expression was knocked down accumulated longer and more unsaturated side chains in neutral ether lipids at the expense of smaller saturated side chains. This was also observed in TGs as well as their *sn*-1 ether-linked counterparts (TG[O/P]), in DGs and CEs. The accumulation was especially pronounced in TGs, where 46 of 300 analyzed species carried a larger and more unsaturated fatty acid signature (supplemental Table S2), whereas 92 of 300 species with shorter more saturated side chains were decreased. Such molecular changes in TG were described in lipidomic analyses of human white adipose tissue samples of which tissues from obese origin contained more polyunsaturated species than samples derived from lean individuals, which tended to accumulate saturated or monounsaturated TGs (46). In our study, we observed a similar effect in in vitro differentiated adipocytes but only by knocking down *Agmo*. Yet, the consequences of obesity on AGMO and ether lipid catabolism are still not clear and direct to future investigations.

For ether-linked phospholipids, it is known that they preferentially carry long side chains with multiple double bonds at their *sn-2* position (47). So far, there is no experimental evidence that ether lipids, especially 1-*O*-alkyl lipids, might serve as precursors for neutral lipid synthesis, and it cannot be easily assumed that remodeling of the massive amounts of TGs present in mature adipocytes would require the much less abundant ether lipids as intermediates. Still, the highly significant impact of *Agmo* knockdown on the lipid architecture of neutral ester lipids points toward a mechanistic requirement for an intact AGMO enzyme for lipid remodeling and exchange of side chains during adipogenesis. Many of the lipids present in our cells share a common structure, but the vast number of possible combinations of carbon chain lengths, unsaturation, and the polar headgroups, as in phospholipids, shape the physicochemical characteristics and create a multiplicity of lipid species (48). They are either synthesized de novo or remodeled from other lipid species, and AGMO might be a crucial part of this remodeling machinery.

To clarify if defective ether lipid degradation would influence genes involved in ether lipid synthesis, we measured expression of *Gnpat*, *Agps*, and *Far1*, the first crucial lipogenic and—in the case of *Far1*—rate-limiting enzymes of ether lipid biosynthesis. We observed no differences in gene expression between the *Agmo* knockdown and the control cell line. Like the analyzed late adipocyte markers and prominent lipolytic genes, *Gnpat* was significantly induced during adipogenesis in 3T3-L1 cells. This has previously been shown by Hajra *et al.* (49) who demonstrated that almost 50% of total TG arise from the contribution of peroxisomal acyl-dihydroxyacetone phosphate synthesis pathway by GNPAT.

By comparing the lipidome of 3T3-L1 adipocytes with compromised or intact AGMO activity, we were able to identify 40 lipid classes and overall more than 2,000 individual lipid species including 179 1-*O*-alkyl and 180 1-*O*-alkenyl species that could be unequivocally assigned. We could show that during an 11-day adipogenic protocol, neutral lipids strongly accumulated, making up more than two-thirds of the lipidome of a mature adipocyte. However, we could not detect many pronounced differences in the overall lipid abundances between *Agmo* knockdown and control cells. Interestingly, the depletion of phosphatidylserine during the course of adipocyte differentiation was significantly compromised upon *Agmo* knockdown but cannot be explained easily. Generally, the behavior of ether lipids and their metabolism during the complex remodeling process happening during adipogenesis is still only poorly understood, and little information is available in the literature. A previous study focusing on lipid profiling in 3T3-L1 preadipocytes had already shown that selected monoalkyldiacylglycerols strongly accumulate toward the end of differentiation (32), a finding that we could confirm in our control cell line. The same study also showed that differentiating cells tend to store almost completely saturated fatty acid side chains with less than 50 carbon atoms in fatty acyl chains in TG. This was also confirmed in the control sh*Luc* adipocytes.

In *Caenorhabditis elegans agmo*-1 mutant worms, it was found that AGMO deficiency led to overall changes in the cuticle lipid composition compared with wild-type strains (19). Mutant worms were viable but had a more diverse lipid profile including accumulation of lipid species with longer side chains, an effect similarly observed in the sh*Agmo* 3T3-L1 adipocytes. These changes in the worm lipidome impacted on the composition of the cuticle, buoyancy, and also resistance of the animal against certain bacterial infections.

In the 3T3-L1 *Agmo* knockdown cells, we observed that plasmalogen (lyso-)phospholipids (both (L)PC[P] and (L)PE[P]) accumulated at day 11 of differentiation when compared with control cells while the respective ester phospholipids were reduced (Fig. 5). The idea of such a phospholipid homeostasis was already proposed by Dorninger *et al.* (50) in 2015 by describing that the total sum of PE lipids (ester and ether) in fibroblasts of rhizomelic chondrodysplasia punctata patients as well as in brains of *Gnpat*-deficient mice was kept constant.

In one of our earlier studies on *Agmo* in RAW264.7 macrophages, we showed that *Agmo* knockdown led to accumulation of 1-*O*-alkyl and 1-*O*-alkenyl phospholipids, whereas glycosylated ceramides and cardiolipins were markedly decreased (17). In our current study on 3T3-L1 adipocyte differentiation, we could also observe accumulation of PE[P] and PC[P] species and their respective lyso-forms upon *Agmo* knockdown (Fig. 5). For cardiolipins, only the lyso-forms were significantly reduced in mature adipocytes, whereas glycosylated ceramides were not influenced at all upon *Agmo* knockdown in our 3T3-L1 differentiation model (Fig. 5). This discrepancy can most likely be attributed to the differences in MS data acquisition quality and subsequent analysis. In our present study, we could rely on recent advances in high-resolution lipidomics and the analysis pipelines that are nowadays able to distinguish between the many often isobaric lipids present in an extract. In our previous study, we had to perform a cluster analysis and lipid subclass enrichment because peaks could not be attributed unequivocally to a defined lipid. A further discrepancy with former data is that in a study by Fischer *et al.* (51), which employed pharmacological inhibition of AGMO by Cp6, a structural homologue to tetrahydrobiopterin, adipocyte differentiation of 3T3-L1 cells and also M2 macrophage polarization in RAW264.7 cells could be inhibited. The basis of this discrepancy is not clear, but 3T3-L1 cells were treated with a relatively high concentration (up to 1 mM) of Cp6, and it cannot be ruled out that this compound also affects other targets besides AGMO.

A most surprising feature of our findings was that *Agmo* knockdown could strongly alter the side-chain composition of the massive amounts of ester-linked triglycerides formed during adipogenesis, although these compounds are no known substrates of *Agmo*.

A clear explanation for the observed effects on ester lipid architecture and plasmalogen levels by reduced ether lipid degradation, in which AGMO is involved in, cannot be easily made according to our current knowledge. However, the AGMO-dependent degradation of plasmanyl lipids clearly contributes to the regulation of the molecular composition of a series of other lipid classes (Fig. 4 and supplemental Fig. S4–S6), especially with regard to their degree of desaturation, therewith potentially involving this catabolic pathway into the homeostasis of a series of related physicochemical properties such as membrane fluidity and susceptibility to lipid peroxidation.

## Data availability

Relative abundances of lipid classes and single metabolites can be found in supplemental data 1 and 2. The raw mass spectrometric data will be made available upon reasonable request (Institute of Biological Chemistry, Biocenter, Medical University of Innsbruck; katrin.watschinger@i-med.ac.at).

## Supplemental data

This article contains supplemental data.

## Conflict of interest

The authors declare that they have no conflicts of interest with the contents of this article.
