# Supplementary material for: Adaptations of the 3T3-L1 adipocyte lipidome to defective ether lipid catabolism upon Agmo knockdown
Source: J Lipid Res. 2022 May 7;63(6):100222. doi: 10.1016/j.jlr.2022.100222 (PMC9192799; doi:10.1016/j.jlr.2022.100222)
Supplement: Supplemental Figures S1–S8 and Tables S1–S6 [file mmc1.docx]

SUPPLEMENTAL INFORMATION:

# **Adaptations of the 3T3-L1 adipocyte lipidome to defective ether lipid catabolism upon *Agmo* knockdown**

Sabrina Sailer^1^, Katharina Lackner^1^, Mia L. Pras-Raves^2,3,4^, Eric J.M. Wever^2,3,4^, Jan B. van Klinken^2,3^, Adriaan D. Dane^2,3,4^, Stephan Geley^5^, Jakob Koch^6^, Georg Golderer^1^, Gabriele Werner-Felmayer^1^, Markus A. Keller^6^, Werner Zwerschke^7^, Frédéric M. Vaz^2,3,8^, Ernst R. Werner^1^, Katrin Watschinger^1#^

^1^Institute of Biological Chemistry, Biocenter, Medical University of Innsbruck, Innsbruck, Austria; ^2^Laboratory Genetic Metabolic Diseases, Department of Clinical Chemistry and Pediatrics, Emma Children's Hospital, Amsterdam UMC Location University of Amssterdam, Amsterdam, The Netherlands; ^3^Core Facility Metabolomics, Amsterdam UMC Location University of Amsterdam, Amsterdam, The Netherlands; ^4^Bioinformatics Laboratory, Department of Epidemiology & Data Science, Amsterdam Public Health Research Institute, Amsterdam UMC Location University of Amsterdam, Amsterdam, The Netherlands; ^5^Institute of Molecular Pathophysiology, Biocenter, Medical University of Innsbruck, Innsbruck, Austria; ^6^Institute of Human Genetics, Medical University of Innsbruck, Innsbruck, Austria; ^7^Division of Cell Metabolism and Differentiation Research, Research Institute for Biomedical Aging Research, University of Innsbruck, Innsbruck, Austria; ^8^Amsterdam Gastroenterology Endocrinology Metabolism, Inborn errors of metabolism, Amsterdam, The Netherlands

# **Supplemental Tables**

| **Gene symbol** | **sense** | **antisense** | **probe** |
| --- | --- | --- | --- |
| ***18S*** | 5’-CCATTCGAACGTCTGCCCTAT-3’ | 5’-TCACCCGTGGTCACCATG-3’ | 5’-ACTTTCGATGGTAGTCGCCGTGCCT-3’ |
| ***Agmo*** | 5’-CTTTCTTAGGAGTTGACTTTGGCTACT-3’ | 5’-TGTGCTGCCCAGAAAATATTAATC-3’ | 5’-CTGGTTCCACCGCATGGCTCATG-3’ |
| ***Adipoq*** | 5’-ATGTTGGAATGACAGGAGCTGAA-3’ | 5’-CACACTGAACGCTGAGCGATA-3’ | 5’-AAAGGAGAGCCTGGAGAAGCCGCTT-3’ |
| ***Agps*** | 5’-AGAGAAATTGCAAAGCAGAGATGTG-3’ | 5’-GAGCATGACCAAACTGGAACTG-3’ | 5’-CTGCATCTATCCGCCTCATGGAC-3’ |
| ***Elovl3*** | 5'-TCTCTTTCTTCTCAGCAAGGTTGTT-3' | 5'-GGACAAAGATGAGTGGACGCTTA-3' | 5'-ACTGGGAGACACGGCCTTCATCATCC-3' |
| ***Far1*** | 5'-CATAAGGCCCCAGCATTCCT-3' | 5'-GAAGACGAGTGATTGTTTTCATCATC-3' | 5'-ACCTCAGGATGACTGGAAGAAGCCCAA-3' |
| ***Fabp4*** | 5’-CTGGGCGTGGAATTCGAT-3’ | 5’-CCCGCCATCTAGGGTTATGA-3’ | 5’-CTCTTCACCTTCCTGTCGTCTGCGGT-3’ |
| ***Fasn*** | 5’-CTCTGAGGACACCGTGACCAT-3’ | 5’-TCTTGCTTTAGCTGCTCCACAA-3’ | 5’-TTCATTCACTGCAGCCTGAGGTCCAG-3’ |
| ***Gnpat*** | 5’-AGGAAGTTCACCCGTCAGCTT-3’ | 5’-GCATTTTTCTGCAGTTCAGAAGAC-3’ | 5’-CAAGGCTCCTCTCAGTGTTACGATGCG-3’ |
| ***Lpl*** | 5’-GGCCGAGAGCGAGAACATT-3’ | 5’-CCGTGTAAATCAAGAAGGAGTAGGTT-3’ | 5’-TTCACCCTGCCCGAGGTTTCCA-3’ |
| ***Lep*** | 5’-ATTTCACACACGCAGTCGGTAT-3’ | 5’-GGTGAAGCCCAGGAATGAAGT-3’ | 5’-CGCCAAGCAGAGGGTCACTGGCT-3’ |
| ***Mgll*** | 5’-TGCTGGTATTTGCCCATGAC-3’ | 5’-CCGACACCACCATCCTCTCT-3’ | 5’-TGTTGGCCATGGGCAGAGTGAGG-3’ |
| ***Pnpla2*** | 5’-CAGCACATTTATCCCGGTGTAC-3’ | 5’-AAATGCCGCCATCCACAT-3’ | 5’-TGGCCTCATTCCTCCTACCCTCCAA-3’ |
| ***Pparg*** | 5’-CACAATGCCATCAGGTTTGG-3’ | 5’-GCTGGTCGATATCACTGGAGATC-3’ | 5’-CCGCCAACAGCTTCTCCTTCTCGG-3’ |

**Supplemental Table S1. Primer and Taqman probe list.**

**
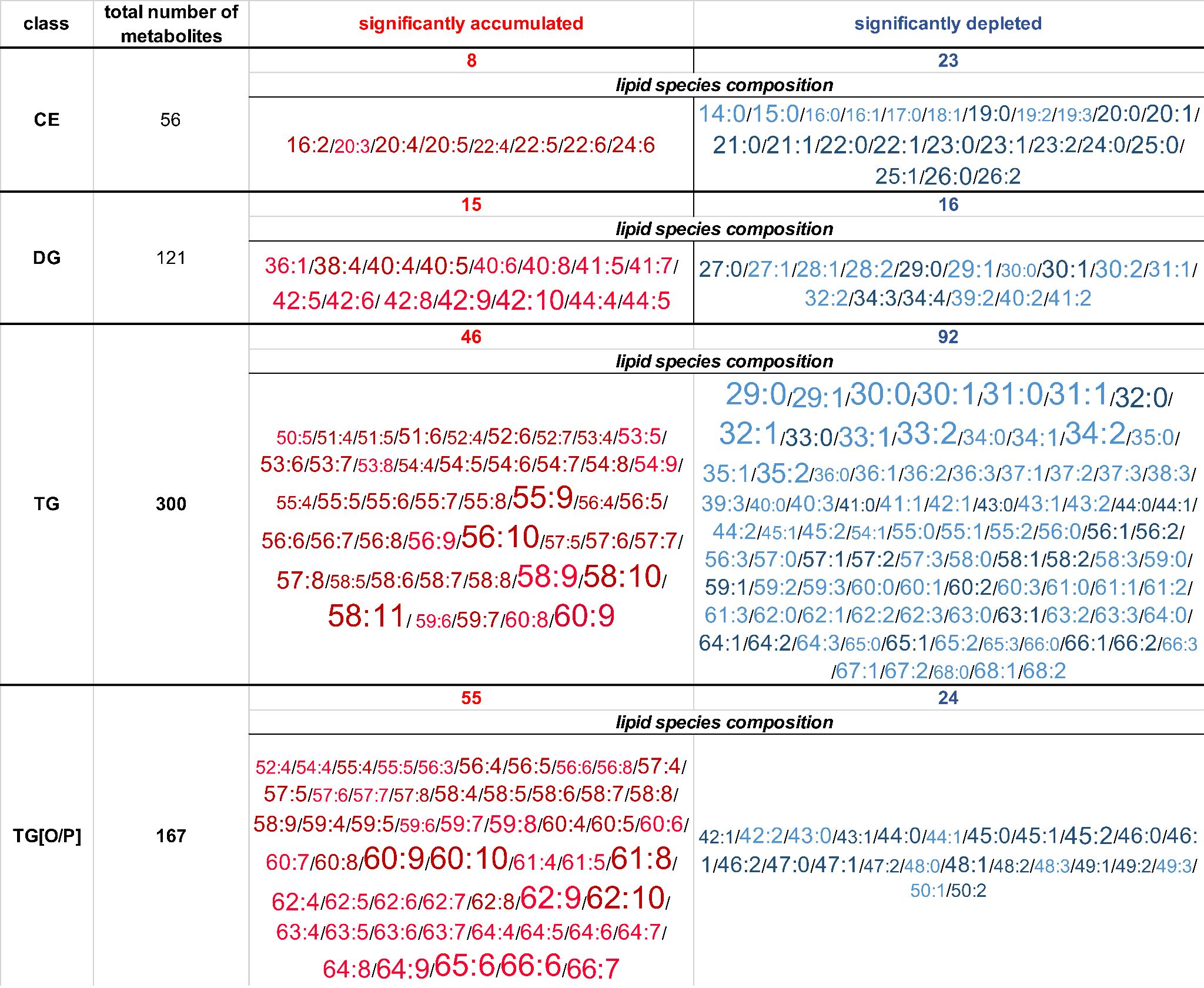
**

**Supplemental Table S2: Significantly accumulated or depleted lipid species in sh*Agmo* adipocytes compared to the sh*Luc* control at day 11 of differentiation.**

The size indicates the fold-change of individual lipid species and the color is representative for the level of significance (light red/blue, *P* < 0.01 and dark red/blue, *P* < 0.001).

| **TG** | **sh*Luc*** | | **sh*Agmo*** | |  |
| --- | --- | --- | --- | --- | --- |
| ***species*** | *mean* | *SD* | *mean* | *SD* | *p-value* |
|  |  |  |  |  |  |
| **TG(29:0)** | 4.29 | 1.30 | 1.20 | 0.49 | 0.001088 |
| **TG(29:1)** | 1.91 | 0.69 | 0.63 | 0.35 | 0.00629 |
| **TG(30:0)** | 19.60 | 6.75 | 5.49 | 1.49 | 0.001843 |
| **TG(30:1)** | 9.90 | 4.24 | 2.46 | 1.18 | 0.005409 |
| **TG(31:0)** | 52.37 | 16.81 | 13.72 | 4.72 | 0.001123 |
| **TG(31:1)** | 33.34 | 13.12 | 7.43 | 3.72 | 0.002798 |
| **TG(32:0)** | 200.35 | 54.41 | 67.02 | 20.13 | 0.000886 |
| **TG(32:1)** | 157.92 | 59.51 | 38.60 | 17.05 | 0.00258 |
| **TG(33:0)** | 386.53 | 81.18 | 167.24 | 46.38 | 0.000779 |
| **TG(33:1)** | 432.54 | 138.20 | 130.75 | 57.39 | 0.001977 |
| **TG(33:2)** | 61.20 | 23.84 | 16.27 | 8.41 | 0.004092 |
| **TG(34:0)** | 734.59 | 145.06 | 376.52 | 84.43 | 0.001408 |
| **TG(34:1)** | 1154.94 | 325.48 | 446.00 | 162.59 | 0.002422 |
| **TG(34:2)** | 288.62 | 107.43 | 87.90 | 37.35 | 0.004258 |
| **TG(35:0)** | 797.63 | 138.33 | 443.11 | 105.72 | 0.001866 |
| **TG(35:1)** | 1447.66 | 348.54 | 666.83 | 224.64 | 0.002953 |
| **TG(35:2)** | 555.40 | 158.07 | 209.38 | 91.99 | 0.002874 |
| **TG(36:0)** | 894.38 | 166.79 | 579.13 | 104.49 | 0.00717 |
| **TG(36:1)** | 1768.12 | 459.97 | 902.48 | 271.68 | 0.00675 |
| **TG(36:2)** | 849.32 | 258.50 | 398.70 | 146.07 | 0.009451 |
| **TG(36:3)** | 28.52 | 8.34 | 12.30 | 4.10 | 0.00454 |
| **TG(37:1)** | 1095.01 | 233.28 | 604.39 | 186.37 | 0.006272 |
| **TG(37:2)** | 533.78 | 123.56 | 281.72 | 99.48 | 0.007475 |
| **TG(37:3)** | 38.69 | 7.85 | 18.11 | 5.49 | 0.00135 |
| **TG(38:3)** | 49.48 | 12.49 | 25.25 | 6.85 | 0.005212 |
| **TG(39:3)** | 29.39 | 5.70 | 15.93 | 4.13 | 0.002698 |
| **TG(40:0)** | 248.16 | 35.74 | 170.96 | 27.81 | 0.005151 |
| **TG(40:3)** | 35.63 | 8.66 | 19.17 | 5.09 | 0.006365 |
| **TG(41:0)** | 225.12 | 17.45 | 150.96 | 24.31 | 0.000547 |
| **TG(41:1)** | 447.38 | 96.95 | 263.29 | 70.23 | 0.008841 |
| **TG(42:1)** | 789.16 | 176.65 | 426.31 | 95.35 | 0.003727 |
| **TG(43:0)** | 271.63 | 8.43 | 220.49 | 13.91 | 0.000109 |
| **TG(43:1)** | 899.83 | 150.75 | 513.36 | 110.24 | 0.001694 |
| **TG(43:2)** | 466.45 | 136.44 | 228.69 | 69.44 | 0.008409 |
| **TG(44:0)** | 421.88 | 16.14 | 361.45 | 15.26 | 0.000295 |
| **TG(44:1)** | 1611.52 | 189.05 | 1071.02 | 145.69 | 0.000972 |
| **TG(44:2)** | 1228.97 | 318.83 | 610.82 | 161.95 | 0.004773 |
| **TG(45:1)** | 1603.37 | 95.76 | 1292.87 | 121.00 | 0.002004 |
| **TG(45:2)** | 1570.86 | 314.21 | 946.50 | 229.53 | 0.007106 |
| **TG(50:5)** | 51.69 | 4.40 | 70.18 | 7.17 | 0.001171 |
| **TG(51:4)** | 67.52 | 5.26 | 88.53 | 6.91 | 0.000639 |
| **TG(51:5)** | 39.68 | 3.62 | 65.47 | 7.82 | 0.000153 |
| **TG(51:6)** | 24.02 | 2.50 | 40.20 | 5.24 | 0.00025 |
| **TG(52:4)** | 91.17 | 4.70 | 118.28 | 12.28 | 0.001733 |
| **TG(52:6)** | 60.91 | 5.19 | 107.40 | 13.03 | 0.000075 |
| **TG(52:7)** | 34.29 | 3.34 | 54.74 | 6.72 | 0.000291 |
| **TG(53:4)** | 41.68 | 3.15 | 57.37 | 5.89 | 0.00077 |
| **TG(53:5)** | 31.60 | 1.95 | 52.92 | 7.07 | 0.000187 |
| **TG(53:6)** | 34.16 | 2.81 | 61.63 | 8.30 | 0.000112 |
| **TG(53:7)** | 27.04 | 3.30 | 47.86 | 6.12 | 0.000153 |
| **TG(53:8)** | 5.75 | 0.83 | 9.03 | 1.45 | 0.002325 |
| **TG(54:1)** | 283.50 | 35.94 | 201.50 | 36.27 | 0.007077 |
| **TG(54:4)** | 60.25 | 3.57 | 86.60 | 7.02 | 0.00007 |
| **TG(54:5)** | 50.31 | 3.13 | 85.96 | 11.24 | 0.000133 |
| **TG(54:6)** | 53.73 | 3.55 | 94.65 | 14.31 | 0.000258 |
| **TG(54:7)** | 43.03 | 3.83 | 77.79 | 12.46 | 0.000338 |
| **TG(54:8)** | 21.62 | 2.14 | 39.80 | 5.20 | 0.000089 |
| **TG(54:9)** | 1.08 | 0.25 | 2.30 | 0.52 | 0.001522 |
| **TG(55:0)** | 53.93 | 9.95 | 28.21 | 8.40 | 0.00224 |
| **TG(55:1)** | 172.27 | 29.91 | 90.86 | 22.93 | 0.001306 |
| **TG(55:2)** | 156.71 | 30.89 | 84.41 | 18.96 | 0.002111 |
| **TG(55:4)** | 23.60 | 2.18 | 32.03 | 2.93 | 0.000852 |
| **TG(55:5)** | 16.34 | 1.14 | 27.56 | 3.49 | 0.000134 |
| **TG(55:6)** | 15.10 | 0.99 | 27.90 | 4.18 | 0.000158 |
| **TG(55:7)** | 13.28 | 1.58 | 25.43 | 3.36 | 0.000083 |
| **TG(55:8)** | 4.83 | 0.83 | 10.65 | 1.56 | 0.000079 |
| **TG(55:9)** | 0.25 | 0.13 | 1.05 | 0.13 | 0.000011 |
| **TG(56:0)** | 56.32 | 9.52 | 28.92 | 8.19 | 0.001221 |
| **TG(56:1)** | 207.89 | 35.40 | 101.63 | 23.60 | 0.000519 |
| **TG(56:2)** | 205.39 | 42.03 | 97.48 | 20.48 | 0.000863 |
| **TG(56:3)** | 90.47 | 17.82 | 54.33 | 7.72 | 0.003158 |
| **TG(56:4)** | 31.04 | 2.66 | 42.60 | 3.53 | 0.000384 |
| **TG(56:5)** | 22.60 | 0.46 | 40.14 | 4.29 | 0.000017 |
| **TG(56:6)** | 19.44 | 1.14 | 34.39 | 5.54 | 0.000358 |
| **TG(56:7)** | 14.57 | 0.70 | 26.89 | 4.46 | 0.000288 |
| **TG(56:8)** | 6.78 | 0.42 | 12.95 | 1.75 | 0.00006 |
| **TG(56:9)** | 1.25 | 0.37 | 3.13 | 0.79 | 0.001317 |
| **TG(56:10)** | 0.33 | 0.15 | 1.24 | 0.27 | 0.000181 |
| **TG(57:0)** | 22.49 | 3.90 | 11.92 | 3.51 | 0.001981 |
| **TG(57:1)** | 97.28 | 19.05 | 45.34 | 11.72 | 0.000829 |
| **TG(57:2)** | 100.50 | 21.81 | 43.60 | 10.74 | 0.000789 |
| **TG(57:3)** | 36.67 | 8.70 | 18.19 | 3.94 | 0.002533 |
| **TG(57:5)** | 7.33 | 0.54 | 10.98 | 1.44 | 0.000711 |
| **TG(57:6)** | 4.76 | 0.40 | 8.69 | 1.19 | 0.000112 |
| **TG(57:7)** | 3.40 | 0.30 | 6.39 | 0.75 | 0.000034 |
| **TG(57:8)** | 0.95 | 0.18 | 2.31 | 0.25 | 0.000009 |
| **TG(58:0)** | 14.17 | 2.57 | 7.91 | 2.29 | 0.003588 |
| **TG(58:1)** | 89.94 | 15.76 | 42.33 | 10.02 | 0.000454 |
| **TG(58:2)** | 116.02 | 21.46 | 54.01 | 11.75 | 0.000472 |
| **TG(58:3)** | 50.82 | 11.35 | 24.18 | 4.52 | 0.001228 |
| **TG(58:5)** | 9.37 | 0.61 | 14.80 | 1.72 | 0.000161 |
| **TG(58:6)** | 7.44 | 0.38 | 12.56 | 1.24 | 0.000022 |
| **TG(58:7)** | 4.18 | 0.17 | 6.95 | 1.01 | 0.000308 |
| **TG(58:8)** | 1.69 | 0.16 | 3.67 | 0.73 | 0.000352 |
| **TG(58:9)** | 0.35 | 0.22 | 1.53 | 0.52 | 0.001646 |
| **TG(58:10)** | 0.31 | 0.20 | 1.71 | 0.29 | 0.000021 |
| **TG(58:11)** | 0.24 | 0.13 | 1.43 | 0.41 | 0.000257 |
| **TG(59:0)** | 4.87 | 0.87 | 2.80 | 0.82 | 0.004767 |
| **TG(59:1)** | 27.09 | 5.31 | 12.70 | 3.35 | 0.000905 |
| **TG(59:2)** | 35.28 | 8.16 | 15.43 | 3.88 | 0.001173 |
| **TG(59:3)** | 15.47 | 3.70 | 7.30 | 1.69 | 0.002023 |
| **TG(59:6)** | 1.89 | 0.22 | 2.64 | 0.43 | 0.008036 |
| **TG(59:7)** | 0.84 | 0.13 | 1.48 | 0.16 | 0.000107 |
| **TG(60:0)** | 3.15 | 0.50 | 1.92 | 0.57 | 0.006761 |
| **TG(60:1)** | 17.51 | 3.34 | 8.76 | 2.06 | 0.001071 |
| **TG(60:2)** | 31.06 | 5.97 | 14.86 | 3.19 | 0.000685 |
| **TG(60:3)** | 18.00 | 3.79 | 8.72 | 1.68 | 0.001053 |
| **TG(60:8)** | 0.49 | 0.13 | 1.01 | 0.20 | 0.001018 |
| **TG(60:9)** | 0.09 | 0.05 | 0.50 | 0.19 | 0.00146 |
| **TG(61:0)** | 1.37 | 0.27 | 0.79 | 0.21 | 0.005233 |
| **TG(61:1)** | 5.17 | 1.06 | 2.48 | 0.70 | 0.001478 |
| **TG(61:2)** | 7.03 | 1.61 | 3.10 | 0.82 | 0.001251 |
| **TG(61:3)** | 3.88 | 0.95 | 1.82 | 0.42 | 0.002204 |
| **TG(62:0)** | 1.16 | 0.25 | 0.66 | 0.17 | 0.005768 |
| **TG(62:1)** | 3.94 | 0.71 | 1.98 | 0.53 | 0.001137 |
| **TG(62:2)** | 5.55 | 1.25 | 2.51 | 0.60 | 0.001179 |
| **TG(62:3)** | 3.54 | 0.75 | 1.82 | 0.38 | 0.001877 |
| **TG(63:0)** | 0.64 | 0.11 | 0.35 | 0.09 | 0.001961 |
| **TG(63:1)** | 1.69 | 0.33 | 0.79 | 0.20 | 0.000875 |
| **TG(63:2)** | 1.67 | 0.40 | 0.78 | 0.20 | 0.002004 |
| **TG(63:3)** | 0.86 | 0.17 | 0.48 | 0.11 | 0.003397 |
| **TG(64:0)** | 0.53 | 0.09 | 0.30 | 0.08 | 0.002541 |
| **TG(64:1)** | 1.56 | 0.29 | 0.74 | 0.16 | 0.000543 |
| **TG(64:2)** | 1.84 | 0.38 | 0.88 | 0.18 | 0.000941 |
| **TG(64:3)** | 1.04 | 0.19 | 0.62 | 0.09 | 0.002175 |
| **TG(65:0)** | 0.29 | 0.03 | 0.18 | 0.03 | 0.001142 |
| **TG(65:1)** | 0.65 | 0.12 | 0.32 | 0.07 | 0.000782 |
| **TG(65:2)** | 0.67 | 0.15 | 0.34 | 0.08 | 0.002207 |
| **TG(65:3)** | 0.37 | 0.06 | 0.25 | 0.03 | 0.005512 |
| **TG(66:0)** | 0.22 | 0.03 | 0.14 | 0.03 | 0.002344 |
| **TG(66:1)** | 0.54 | 0.10 | 0.26 | 0.06 | 0.000722 |
| **TG(66:2)** | 0.68 | 0.13 | 0.34 | 0.07 | 0.00067 |
| **TG(66:3)** | 0.49 | 0.09 | 0.34 | 0.06 | 0.009133 |
| **TG(67:1)** | 0.20 | 0.04 | 0.10 | 0.02 | 0.002945 |
| **TG(67:2)** | 0.20 | 0.04 | 0.12 | 0.03 | 0.007314 |
| **TG(68:0)** | 0.06 | 0.01 | 0.04 | 0.01 | 0.006571 |
| **TG(68:1)** | 0.13 | 0.03 | 0.07 | 0.01 | 0.001151 |
| **TG(68:2)** | 0.16 | 0.03 | 0.09 | 0.01 | 0.001478 |

**Supplemental Table S3: Relative abundances of individual TG species that were significantly different between the sh*Luc* and sh*Agmo* cell line at day 11 of adipocyte differentiation.**

All 138 significantly different lipid species between sh*Luc* and sh*Agmo* mature adipocytes are listed (relative abundance of the total TG pool: sh*Luc* = 71204.35; sh*Agmo* = 57213.11). The corresponding volcano plot is shown in Fig. 4.

| **TG[O/P]** | **sh*Luc*** | | **sh*Agmo*** | |  |
| --- | --- | --- | --- | --- | --- |
| ***species*** | *mean* | *SD* | *mean* | *SD* | *p-value* |
|  |  |  |  |  |  |
| **TG(O-42:1)** | 1.35 | 0.09 | 0.94 | 0.07 | 0.000043 |
| **TG(O-42:2)** | 1.11 | 0.14 | 0.69 | 0.21 | 0.005712 |
| **TG(O-43:0)** | 1.38 | 0.24 | 0.81 | 0.18 | 0.003019 |
| **TG(O-43:1)** | 1.24 | 0.12 | 0.85 | 0.09 | 0.000375 |
| **TG(O-44:0)** | 4.82 | 0.31 | 2.67 | 0.38 | 0.000011 |
| **TG(O-44:1)** | 3.14 | 0.30 | 2.19 | 0.34 | 0.001617 |
| **TG(O-45:0)** | 16.54 | 1.52 | 8.37 | 0.95 | 0.000007 |
| **TG(O-45:1)** | 8.42 | 0.44 | 4.85 | 0.69 | 0.00001 |
| **TG(O-45:2)** | 1.60 | 0.21 | 0.70 | 0.14 | 0.000044 |
| **TG(O-46:0)** | 68.52 | 5.46 | 37.58 | 4.59 | 0.000011 |
| **TG(O-46:1)** | 35.72 | 2.67 | 21.64 | 1.90 | 0.000011 |
| **TG(O-46:2)** | 7.20 | 0.85 | 3.56 | 1.34 | 0.00088 |
| **TG(O-47:0)** | 151.20 | 19.13 | 89.33 | 11.21 | 0.000248 |
| **TG(O-47:1)** | 94.19 | 9.49 | 56.09 | 7.54 | 0.000109 |
| **TG(O-47:2)** | 19.82 | 1.48 | 13.63 | 1.49 | 0.000173 |
| **TG(O-48:0)** | 230.76 | 28.06 | 158.48 | 24.30 | 0.002432 |
| **TG(O-48:1)** | 284.27 | 25.65 | 182.25 | 13.70 | 0.00005 |
| **TG(O-48:2)** | 75.25 | 5.50 | 51.87 | 3.78 | 0.00005 |
| **TG(O-48:3)** | 8.61 | 0.43 | 7.13 | 0.64 | 0.002585 |
| **TG(O-49:1)** | 221.16 | 22.75 | 152.89 | 15.20 | 0.000523 |
| **TG(O-49:2)** | 78.42 | 7.63 | 54.25 | 5.45 | 0.000422 |
| **TG(O-49:3)** | 8.46 | 0.96 | 6.63 | 0.63 | 0.007393 |
| **TG(O-50:1)** | 292.94 | 18.29 | 237.78 | 24.46 | 0.003742 |
| **TG(O-50:2)** | 172.12 | 12.66 | 131.12 | 10.74 | 0.00056 |
| **TG(O-52:4)** | 13.07 | 0.98 | 16.29 | 1.68 | 0.006118 |
| **TG(O-54:4)** | 20.49 | 2.10 | 28.81 | 3.77 | 0.002593 |
| **TG(O-55:4)** | 7.37 | 0.82 | 11.04 | 1.32 | 0.000742 |
| **TG(O-55:5)** | 21.85 | 1.53 | 29.15 | 4.47 | 0.008619 |
| **TG(O-56:3)** | 7.50 | 0.41 | 10.86 | 1.85 | 0.004127 |
| **TG(O-56:4)** | 9.15 | 0.38 | 17.17 | 2.73 | 0.000186 |
| **TG(O-56:5)** | 22.08 | 2.10 | 39.96 | 6.44 | 0.000361 |
| **TG(O-56:6)** | 38.81 | 3.30 | 55.82 | 8.25 | 0.002684 |
| **TG(O-56:8)** | 3.60 | 0.23 | 5.07 | 0.86 | 0.00629 |
| **TG(O-57:4)** | 2.02 | 0.25 | 3.87 | 0.65 | 0.000339 |
| **TG(O-57:5)** | 3.93 | 0.28 | 7.24 | 1.29 | 0.000496 |
| **TG(O-57:6)** | 6.67 | 0.32 | 10.37 | 1.73 | 0.00152 |
| **TG(O-57:7)** | 4.61 | 0.52 | 6.28 | 0.85 | 0.005624 |
| **TG(O-57:8)** | 1.01 | 0.11 | 1.59 | 0.10 | 0.00002 |
| **TG(O-58:4)** | 2.77 | 0.16 | 5.54 | 0.87 | 0.000118 |
| **TG(O-58:5)** | 5.02 | 0.57 | 10.80 | 1.72 | 0.000102 |
| **TG(O-58:6)** | 7.90 | 0.82 | 14.81 | 2.35 | 0.000255 |
| **TG(O-58:7)** | 6.26 | 0.68 | 10.65 | 1.29 | 0.000147 |
| **TG(O-58:8)** | 2.41 | 0.46 | 4.05 | 0.45 | 0.000475 |
| **TG(O-58:9)** | 0.64 | 0.18 | 1.41 | 0.27 | 0.000701 |
| **TG(O-59:4)** | 0.53 | 0.05 | 0.95 | 0.16 | 0.000443 |
| **TG(O-59:5)** | 0.77 | 0.01 | 1.56 | 0.30 | 0.000366 |
| **TG(O-59:6)** | 1.34 | 0.10 | 2.15 | 0.43 | 0.003525 |
| **TG(O-59:7)** | 0.78 | 0.13 | 1.33 | 0.21 | 0.001211 |
| **TG(O-59:8)** | 0.25 | 0.09 | 0.58 | 0.13 | 0.001365 |
| **TG(O-60:4)** | 0.73 | 0.05 | 1.46 | 0.28 | 0.000462 |
| **TG(O-60:5)** | 1.36 | 0.16 | 2.70 | 0.57 | 0.00095 |
| **TG(O-60:6)** | 2.04 | 0.14 | 3.62 | 0.69 | 0.001077 |
| **TG(O-60:7)** | 1.51 | 0.22 | 2.63 | 0.50 | 0.001817 |
| **TG(O-60:8)** | 0.74 | 0.11 | 1.62 | 0.24 | 0.00008 |
| **TG(O-60:9)** | 0.43 | 0.12 | 1.27 | 0.22 | 0.00007 |
| **TG(O-60:10)** | 0.21 | 0.06 | 0.93 | 0.13 | 0.000003 |
| **TG(O-61:4)** | 0.17 | 0.02 | 0.33 | 0.08 | 0.001873 |
| **TG(O-61:5)** | 0.33 | 0.02 | 0.61 | 0.16 | 0.004725 |
| **TG(O-61:8)** | 0.07 | 0.04 | 0.23 | 0.05 | 0.000486 |
| **TG(O-62:4)** | 0.28 | 0.07 | 0.68 | 0.18 | 0.001927 |
| **TG(O-62:5)** | 0.70 | 0.06 | 1.36 | 0.36 | 0.003668 |
| **TG(O-62:6)** | 1.16 | 0.10 | 2.00 | 0.49 | 0.005807 |
| **TG(O-62:7)** | 0.81 | 0.08 | 1.30 | 0.31 | 0.0095 |
| **TG(O-62:8)** | 0.35 | 0.05 | 0.71 | 0.12 | 0.000236 |
| **TG(O-62:9)** | 0.14 | 0.01 | 0.56 | 0.04 | <0,000001 |
| **TG(O-62:10)** | 0.13 | 0.07 | 0.44 | 0.09 | 0.000287 |
| **TG(O-63:4)** | 0.06 | 0.01 | 0.12 | 0.04 | 0.009412 |
| **TG(O-63:5)** | 0.11 | 0.02 | 0.21 | 0.05 | 0.004505 |
| **TG(O-63:6)** | 0.16 | 0.03 | 0.30 | 0.08 | 0.007054 |
| **TG(O-63:7)** | 0.10 | 0.02 | 0.19 | 0.04 | 0.003117 |
| **TG(O-64:4)** | 0.11 | 0.01 | 0.23 | 0.07 | 0.006637 |
| **TG(O-64:5)** | 0.20 | 0.00 | 0.40 | 0.11 | 0.003436 |
| **TG(O-64:6)** | 0.28 | 0.06 | 0.57 | 0.13 | 0.002176 |
| **TG(O-64:7)** | 0.24 | 0.05 | 0.49 | 0.14 | 0.005377 |
| **TG(O-64:8)** | 0.12 | 0.03 | 0.27 | 0.08 | 0.002802 |
| **TG(O-64:9)** | 0.06 | 0.03 | 0.15 | 0.05 | 0.007578 |
| **TG(O-65:6)** | 0.01 | 0.01 | 0.05 | 0.02 | 0.006619 |
| **TG(O-66:6)** | 0.02 | 0.01 | 0.08 | 0.03 | 0.00436 |
| **TG(O-66:7)** | 0.03 | 0.01 | 0.07 | 0.02 | 0.006932 |

**Supplemental Table S4: Relative abundances of individual TG[O/P] species that were significantly different between the sh*Luc* and sh*Agmo* cell line at day 11 of adipocyte differentiation.**

All 79 significantly different species between sh*Luc* and sh*Agmo* mature adipocytes are listed (relative abundance of the total TG[O/P] pool: sh*Luc* = 3172.94; sh*Agmo* = 2738.20). Mean ± SD, n = 5. The corresponding volcano plot is shown in supplemental Fig. S4.

| **DG** | **sh*Luc*** | | **sh*Agmo*** | |  |
| --- | --- | --- | --- | --- | --- |
| ***species*** | *mean* | *SD* | *mean* | *SD* | *p-value* |
|  |  |  |  |  |  |
| **DG(27:0)** | 41.13 | 3.74 | 27.15 | 3.73 | 0.00036 |
| **DG(27:1)** | 44.98 | 6.02 | 31.10 | 6.98 | 0.0099 |
| **DG(28:1)** | 278.8 | 38.8 | 184.7 | 38.7 | 0.00496 |
| **DG(28:2)** | 39.9 | 7.7 | 22.1 | 7.0 | 0.0051 |
| **DG(29:0)** | 467.28 | 16.47 | 345.46 | 46.47 | 0.00056 |
| **DG(29:1)** | 357.48 | 45.00 | 214.98 | 47.55 | 0.0012 |
| **DG(30:0)** | 1606.74 | 75.13 | 1333.33 | 154.08 | 0.0073 |
| **DG(30:1)** | 2778.95 | 355.29 | 1641.56 | 271.66 | 0.00046 |
| **DG(30:2)** | 629.70 | 108.45 | 363.78 | 81.70 | 0.0024 |
| **DG(31:1)** | 5503.25 | 479.52 | 4017.80 | 720.92 | 0.00497 |
| **DG(32:2)** | 1019.99 | 172.33 | 669.83 | 159.46 | 0.0031 |
| **DG(34:3)** | 919.91 | 59.09 | 669.37 | 88.67 | 0.00077 |
| **DG(34:4)** | 175.82 | 12.78 | 123.02 | 17.99 | 0.00068 |
| **DG(36:1)** | 436.58 | 77.54 | 711.64 | 164.33 | 0.0096 |
| **DG(38:4)** | 821.94 | 43.07 | 1521.07 | 200.58 | 0.000062 |
| **DG(39:2)** | 13.08 | 0.84 | 9.44 | 1.86 | 0.004 |
| **DG(40:2)** | 34.21 | 2.78 | 22.72 | 4.79 | 0.0017 |
| **DG(40:4)** | 34.78 | 3.40 | 58.71 | 11.41 | 0.002 |
| **DG(40:5)** | 74.65 | 7.13 | 142.80 | 28.16 | 0.00078 |
| **DG(40:6)** | 61.57 | 6.56 | 89.24 | 16.06 | 0.0073 |
| **DG(40:8)** | 19.04 | 3.50 | 33.23 | 8.23 | 0.0075 |
| **DG(41:2)** | 8.38 | 0.72 | 5.39 | 1.51 | 0.00399 |
| **DG(41:5)** | 2.31 | 0.20 | 4.13 | 0.95 | 0.003 |
| **DG(41:7)** | 3.33 | 0.39 | 4.94 | 0.91 | 0.0068 |
| **DG(42:5)** | 1.96 | 0.38 | 3.79 | 0.80 | 0.0017 |
| **DG(42:6)** | 2.80 | 0.48 | 5.38 | 1.14 | 0.0016 |
| **DG(42:8)** | 2.75 | 0.65 | 6.13 | 1.77 | 0.0039 |
| **DG(42:9)** | 5.29 | 1.16 | 12.69 | 3.80 | 0.0031 |
| **DG(42:10)** | 7.89 | 2.25 | 20.82 | 6.13 | 0.0022 |
| **DG(44:4)** | 0.78 | 0.28 | 1.68 | 0.47 | 0.0066 |
| **DG(44:5)** | 0.22 | 0.18 | 0.57 | 0.32 | 0.0065 |

**Supplemental Table S5: Relative abundances of individual DG species that were significantly different between the sh*Luc* and sh*Agmo* cell line at day 11 of adipocyte differentiation.**

All 31 significantly different species between sh*Luc* and sh*Agmo* mature adipocytes are listed (relative abundance of the total DG pool: sh*Luc* = 71741.89; sh*Agmo* = 64331.31). The corresponding volcano plot is shown in supplemental Fig. S5.

| **CE** | **sh*Luc*** | | **sh*Agmo*** | |  |
| --- | --- | --- | --- | --- | --- |
| ***species*** | *mean* | *SD* | *mean* | *SD* | *p-value* |
|  |  |  |  |  |  |
| **CE(14:0)** | 126.81 | 17.19 | 68.35 | 8.44 | 0.000134 |
| **CE(15:0)** | 601.31 | 43.78 | 350.99 | 64.21 | 0.000092 |
| **CE(16:0)** | 2497.5 | 176.5 | 1668.0 | 234.2 | 0.000226 |
| **CE(16:1)** | 5684.7 | 449.2 | 4258.2 | 801.0 | 0.008404 |
| **CE(16:2)** | 55.69 | 7.04 | 95.55 | 12.53 | 0.000259 |
| **CE(17:0)** | 405.91 | 28.29 | 285.40 | 41.49 | 0.000672 |
| **CE(18:1)** | 7582.62 | 361.37 | 6061.92 | 839.67 | 0.005873 |
| **CE(19:0)** | 40.72 | 3.67 | 25.77 | 6.44 | 0.001978 |
| **CE(19:2)** | 162.47 | 13.98 | 122.34 | 15.45 | 0.002593 |
| **CE(19:3)** | 297.66 | 26.86 | 220.10 | 41.27 | 0.007824 |
| **CE(20:0)** | 92.19 | 10.44 | 54.33 | 15.05 | 0.001708 |
| **CE(20:1)** | 345.10 | 25.47 | 229.83 | 44.86 | 0.001058 |
| **CE(20:3)** | 850.13 | 55.54 | 1058.10 | 117.85 | 0.007299 |
| **CE(20:4)** | 2750.86 | 247.22 | 4247.36 | 509.94 | 0.00036 |
| **CE(20:5)** | 346.46 | 46.30 | 604.11 | 144.55 | 0.005269 |
| **CE(21:0)** | 20.38 | 1.73 | 10.37 | 2.02 | 0.00003 |
| **CE(21:1)** | 48.20 | 7.67 | 25.37 | 5.88 | 0.000743 |
| **CE(22:0)** | 72.60 | 7.66 | 40.71 | 10.20 | 0.000517 |
| **CE(22:1)** | 191.21 | 24.80 | 104.84 | 26.27 | 0.000689 |
| **CE(22:4)** | 166.86 | 13.88 | 224.14 | 26.30 | 0.002591 |
| **CE(22:5)** | 494.75 | 58.17 | 765.05 | 65.84 | 0.000127 |
| **CE(22:6)** | 352.01 | 23.94 | 523.41 | 37.05 | 0.000024 |
| **CE(23:0)** | 25.91 | 3.49 | 13.61 | 3.35 | 0.000461 |
| **CE(23:1)** | 49.81 | 5.01 | 27.14 | 8.61 | 0.000943 |
| **CE(23:2)** | 7.35 | 1.42 | 4.57 | 0.77 | 0.004913 |
| **CE(24:0)** | 177.86 | 24.08 | 106.11 | 28.88 | 0.002738 |
| **CE(24:6)** | 17.70 | 3.40 | 25.22 | 2.79 | 0.005066 |
| **CE(25:0)** | 35.67 | 3.86 | 20.13 | 5.29 | 0.000721 |
| **CE(25:1)** | 44.07 | 3.99 | 26.09 | 7.46 | 0.001446 |
| **CE(26:0)** | 52.58 | 7.25 | 29.28 | 8.60 | 0.001681 |
| **CE(26:2)** | 28.96 | 4.87 | 17.89 | 3.83 | 0.003992 |

**Supplemental Table S6: Relative abundances of individual CE species that were significantly different between the sh*Luc* and sh*Agmo* cell line at day 11 of adipocyte differentiation.**

All 31 significantly different species between sh*Luc* and sh*Agmo* mature adipocytes are listed (relative abundance of the total CE pool: sh*Luc* = 28161.62; sh*Agmo* = 25301.77). The corresponding volcano plot is shown in supplemental Fig. S6.

# **Supplemental Figures**


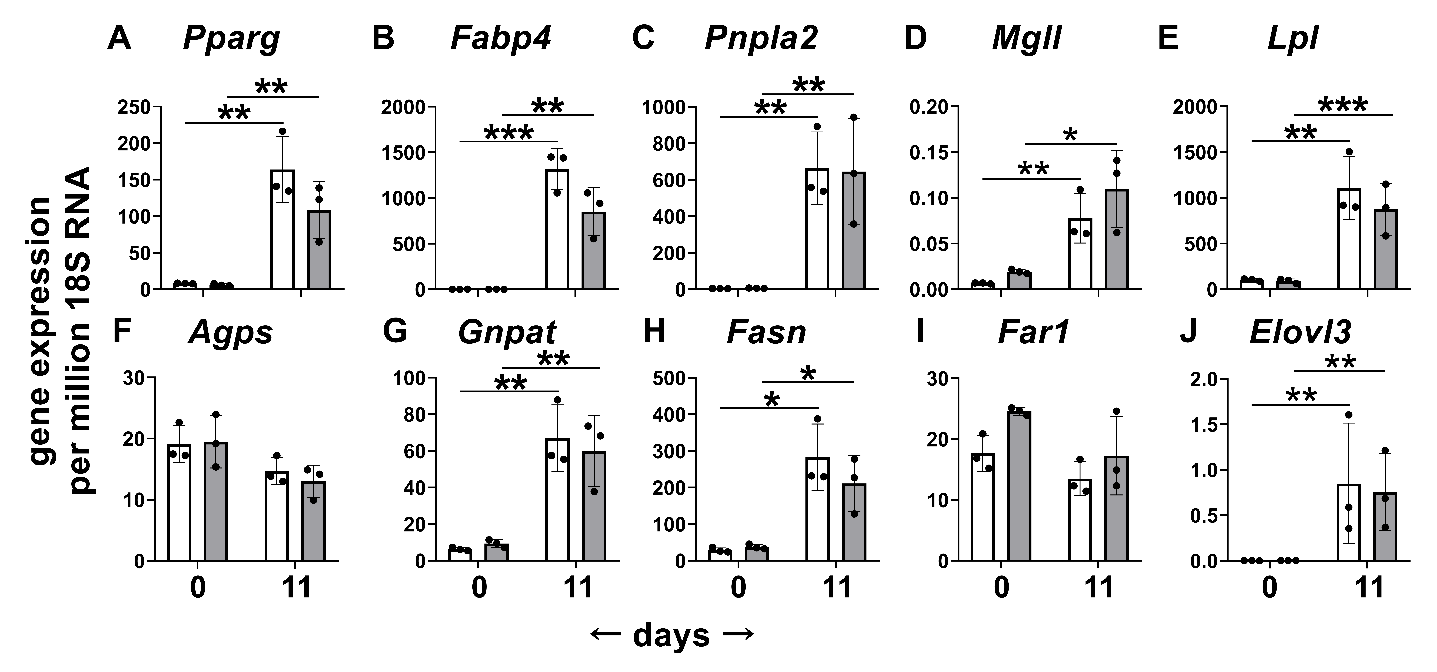


**Supplemental Fig. S1. Effect of *Agmo* knockdown on the expression of selected adipocyte specific, lipogenic and lipolytic genes during adipocyte differentiation at day 0 and day 11 of adipocyte differentiation.** Gene expression analysis was performed by RT-qPCR using Taqman technology. Expression of adipocyte markers **A)** peroxisome proliferator activated receptor gamma (*Pparg*) and **B)** fatty acid binding protein 4 (*Fabp4*). Gene expression analysis of lipolytic genes **C)** patatin-like phospholipase domain-containing protein 2 (*Pnpla2*), **D)** monoacylglycerol lipase (*Mgll*) and **E)** lipoprotein lipase (*Lpl*). Gene expression analysis of lipogenic genes **F)** alkylglycerone phosphate synthase (*Agps*), **G)** glyceronephosphate *O*-acyltransferase (*Gnpat*), **H)** fatty acid synthase (*Fasn*), **I)** fatty acyl-CoA reductase 1 (*Far1*) and **J)** elongation of very long chain fatty acids 3 (*Elovl3*). The sh*Luc* cell line is presented by the open bars and gray bars show the sh*Agmo* cell line (n = 3). Mean ± SEM, * *P <* 0.05, ** *P <* 0.01 and *** *P <* 0.001.


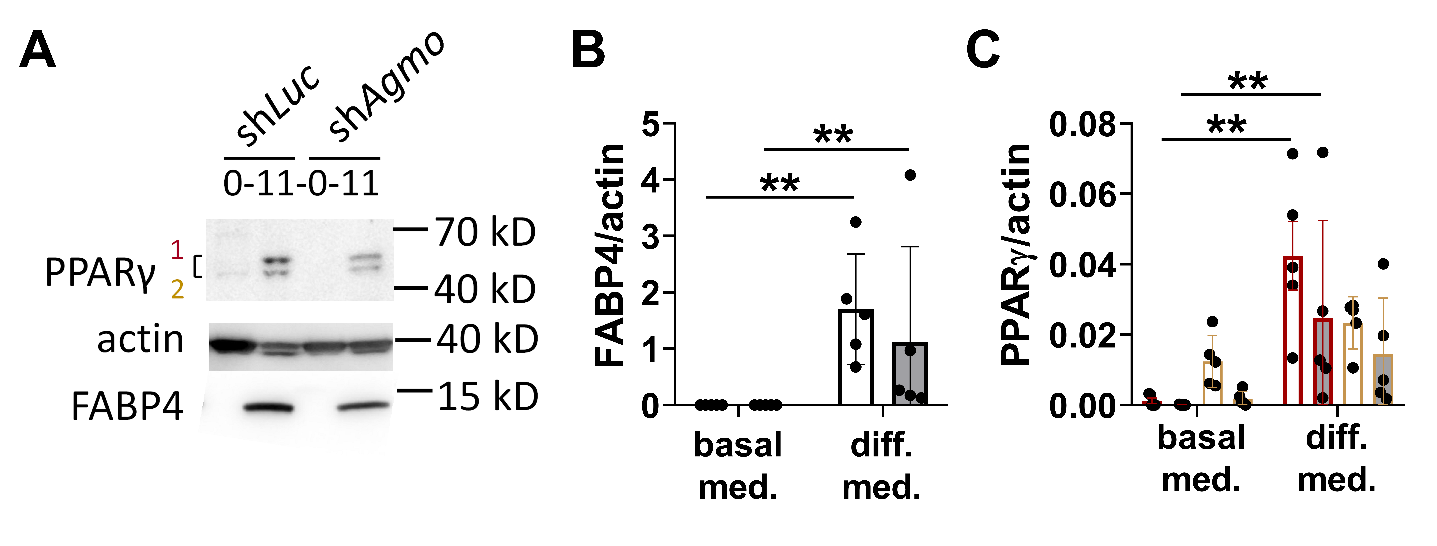


**Supplemental Fig. S2. The influence of *Agmo* knockdown on protein expression of FABP4 and PPARγ (isoforms 1 and 2) during adipocyte differentiation.** Both cell lines, sh*Luc* and sh*Agmo* were harvested at day 0 and day 11 of adipocyte differentiation for assessment of FABP4 and PPARγ protein amount. **A)** Representative Western blot of sh*Luc* and sh*Agmo*. **B)** Densitometric quantification of FABP4 with sh*Luc* in open bars and sh*Agmo* in gray bars is shown. **C)** Densitometric quantification of the PPARγ isoform 1 (outlined in red) and 2 (outlined in beige) with sh*Luc* (open bars) and sh*Agmo* (gray bars) is shown. Mean ± SD, n = 5, ** *P <* 0.01.

.


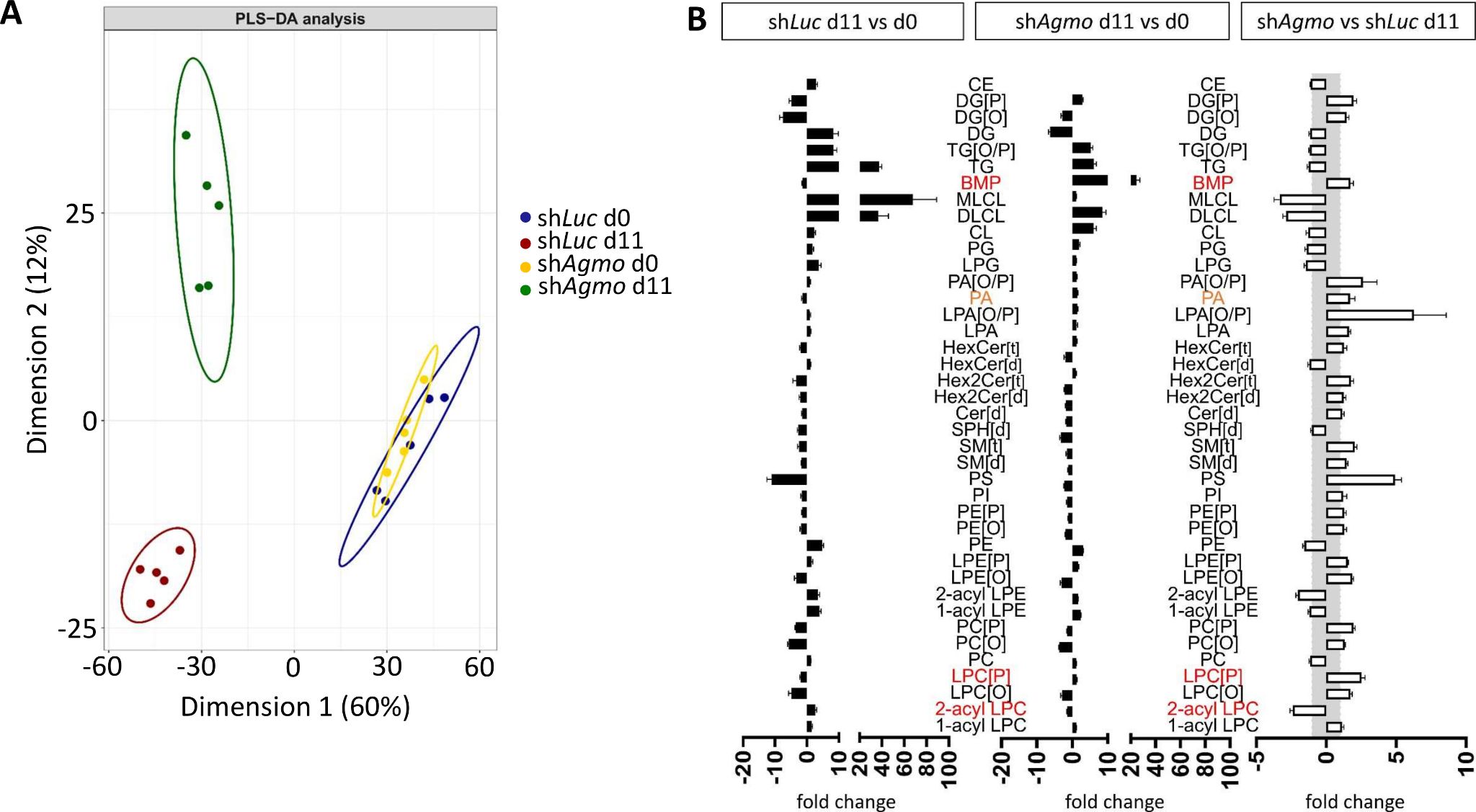


**Supplemental Fig. S3. Partial least squares regression (PLS regression) combined with discriminant analysis (DA) of lipidomics data.** **A)** The five replicates represented by dots colored according to sample group and framed by ellipses are positioned in a multidimensional metabolite space and dimensionality is reduced by projection on the principal plane formed by the first two principal axes (dimension 1 and dimension 2), which cover 60% and 12%, respectively, of the variance. Dimension 1 discriminates in respect to the days of differentiation whereas dimension 2 separates according to cell line differences. Blue shows the sh*Luc* cell line at day 0 and red at day 11 of differentiation. The sh*Agmo* cell line at day 0 is depicted in yellow and in green at day 11 of adipocyte differentiation. **B)** Bar charts presenting the fold changes of lipid abundances during adipocyte differentiation from day 0 to day 11 of the sh*Luc* and sh*Agmo* cell lines. The open bars depict the fold changes between both cell lines at day 11. The gray zone ranges from -1 to 1 and indicates the space where no obvious changes are located. In red, lipid classes are shown that are significantly differentially regulated in the sh*Agmo* knockdown cell line at day 11 compared to the shLuc control cell at day 11. In orange, phosphatidic acid is shown that is differentially regulated in the sh*Agmo* knockdown cell line at day 11 compared to the shLuc control cell at day 11 but did not reach significance. Mean ± SEM, n = 5.

**
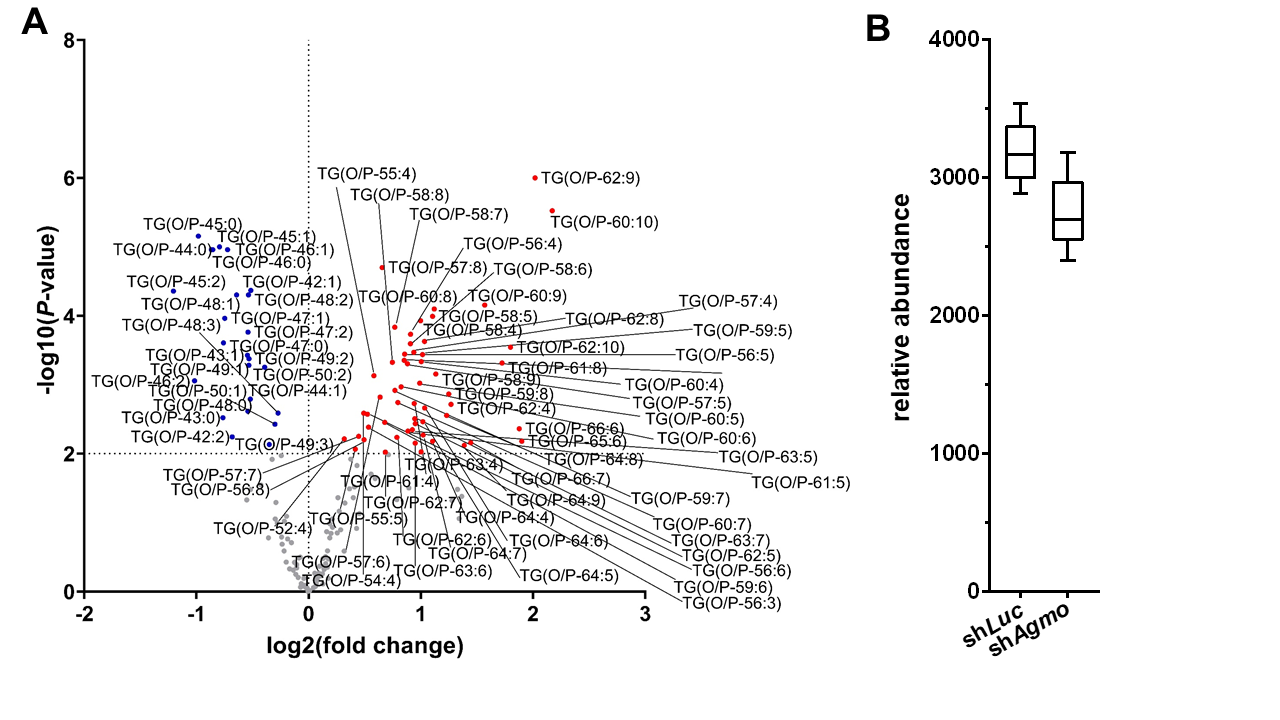
**

**Supplemental Fig. S4. Detailed analysis of carbon chain length and degree of desaturation of single TG[O/P]** **species upon *Agmo* knockdown in mature adipocytes.** **A)** Volcano plot depicting the lipidomics data of TG[O/P] at day 11 of adipocyte differentiation. Signiﬁcance cut-oﬀ is *P* < 0.01 as indicated by the horizontal dotted line. The vertical dotted line separates the log2(fold change) of decreased (blue dots) and increased (red dots) lipid species. **B)** Boxplots showing the relative abundances of TG[O/P] at day 11 of adipocyte differentiation in the sh*Luc* and sh*Agmo* cell lines. An overview about the significantly accumulated or depleted metabolites is shown in supplemental Table S2. In supplemental Table S4 the relative abundances of all significantly changed TG[O/P] species are provided.

**
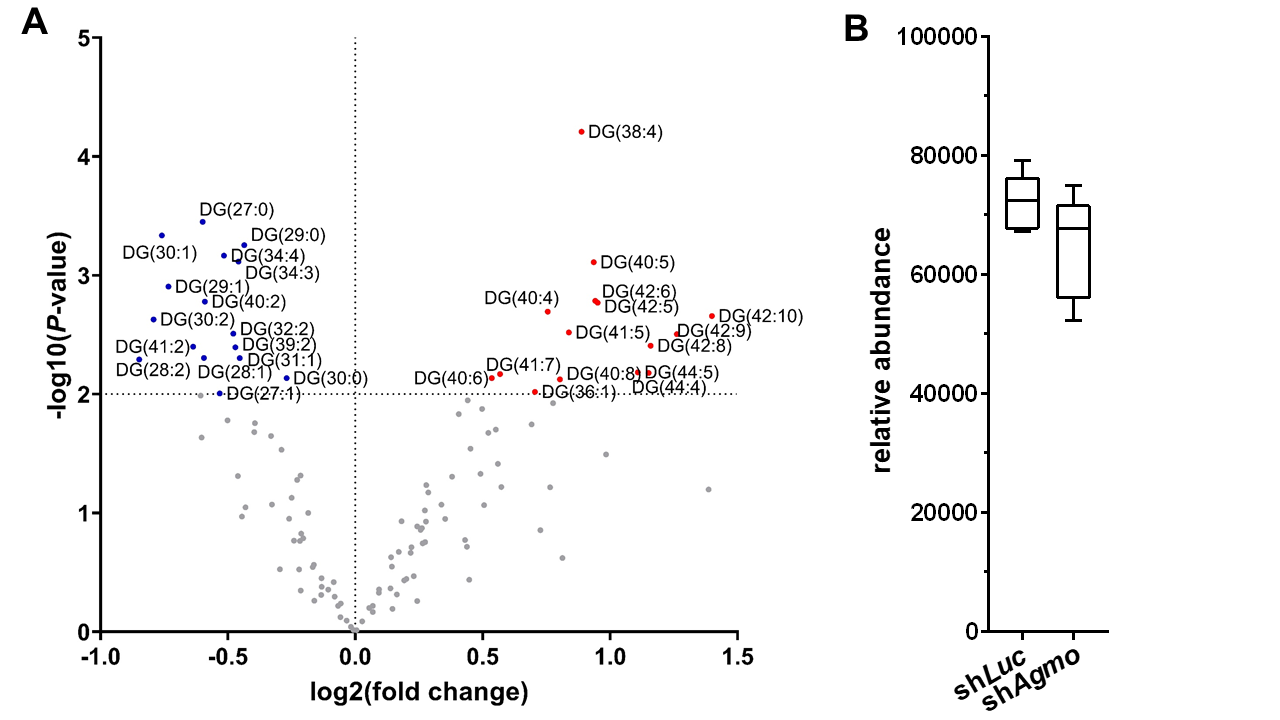
**

**Supplemental Fig. S5. Detailed analysis of carbon chain length and degree of desaturation of single DG species upon *Agmo* knockdown in mature adipocytes.** **A)** Volcano plot depicting the lipidomics data of DG at day 11 of adipocyte differentiation. Signiﬁcance cut-oﬀ is *P* < 0.01 as indicated by the horizontal dotted line. The vertical dotted line separates the log2(fold change) of decreased (blue dots) and increased (red dots) lipid species. **B)** Boxplots showing the relative abundances of DG at day 11 of adipocyte differentiation in the sh*Luc* and sh*Agmo* cell lines. An overview about the significantly accumulated or depleted metabolites is shown in supplemental Table 2. In supplemental Table S5 the relative abundances of all significantly changed DG species are provided.

**
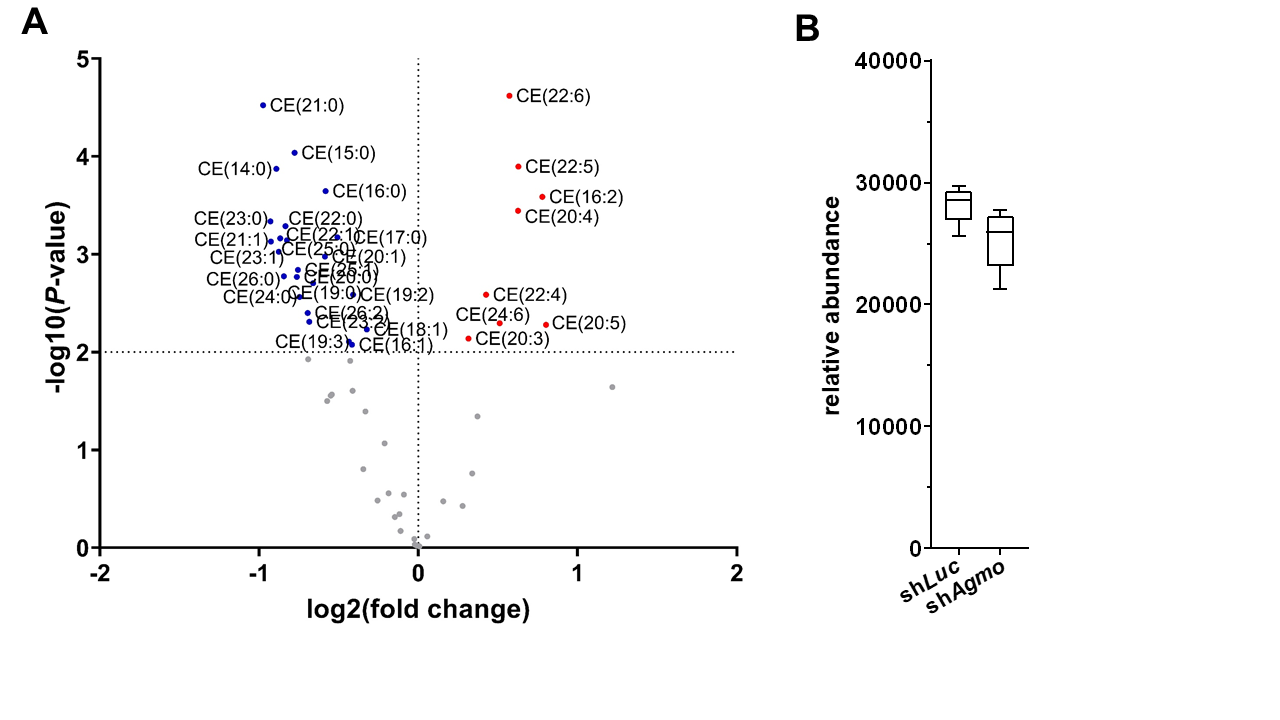
**

**Supplemental Fig. S6. Detailed analysis of carbon chain length and degree of desaturation of single CE species upon *Agmo* knockdown in mature adipocytes.** **A)** Volcano plot depicting the lipidomics data of CE at day 11 of adipocyte differentiation. Signiﬁcance cut-oﬀ is *P* < 0.01 as indicated by the horizontal dotted line. The vertical dotted line separates the log2(fold change) of decreased (blue dots) and increased (red dots) lipid species. **B)** Boxplots showing the relative abundances of CE at day 11 of adipocyte differentiation in the sh*Luc* and sh*Agmo* cell lines. An overview about the significantly accumulated or depleted metabolites is shown in supplemental Table 2. In supplemental Table S6 the relative abundances of all significantly changed CE species are provided.


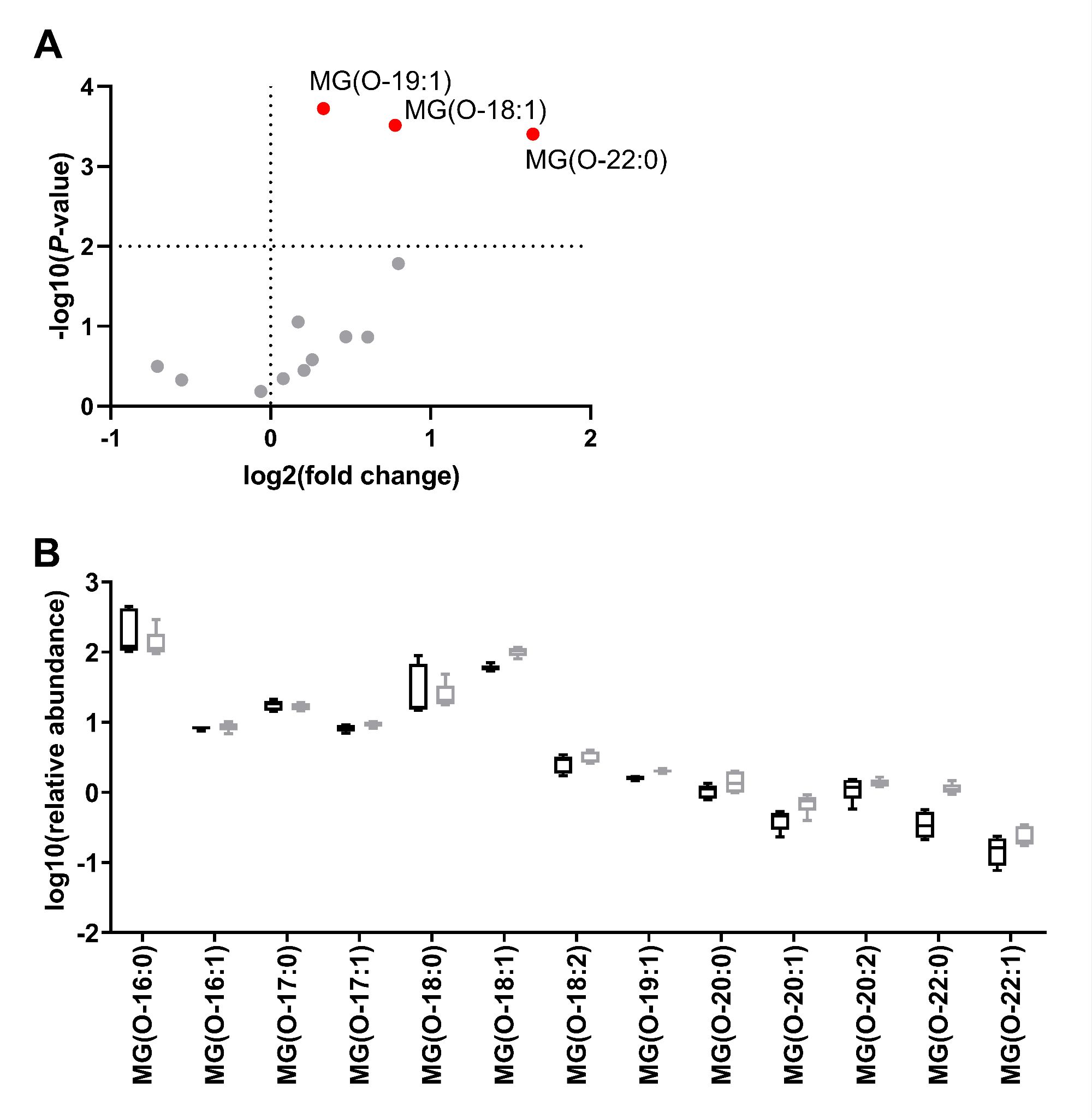


**Supplemental Fig. S7. Detailed analysis of carbon chain length and degree of desaturation of single MG[O]** **species upon *Agmo* knockdown in mature adipocytes.** **A)** Volcano plot depicting the lipidomics data of MG[O] at day 11 of adipocyte differentiation. Signiﬁcance cut-oﬀ is *P* < 0.01 as indicated by the horizontal dotted line. The vertical dotted line separates the log2(fold change) of decreased and increased (red dots) lipid species. **B)** Boxplots showing the relative abundances of MG[O] at day 11 of adipocyte differentiation in the sh*Luc* and sh*Agmo* cell lines.


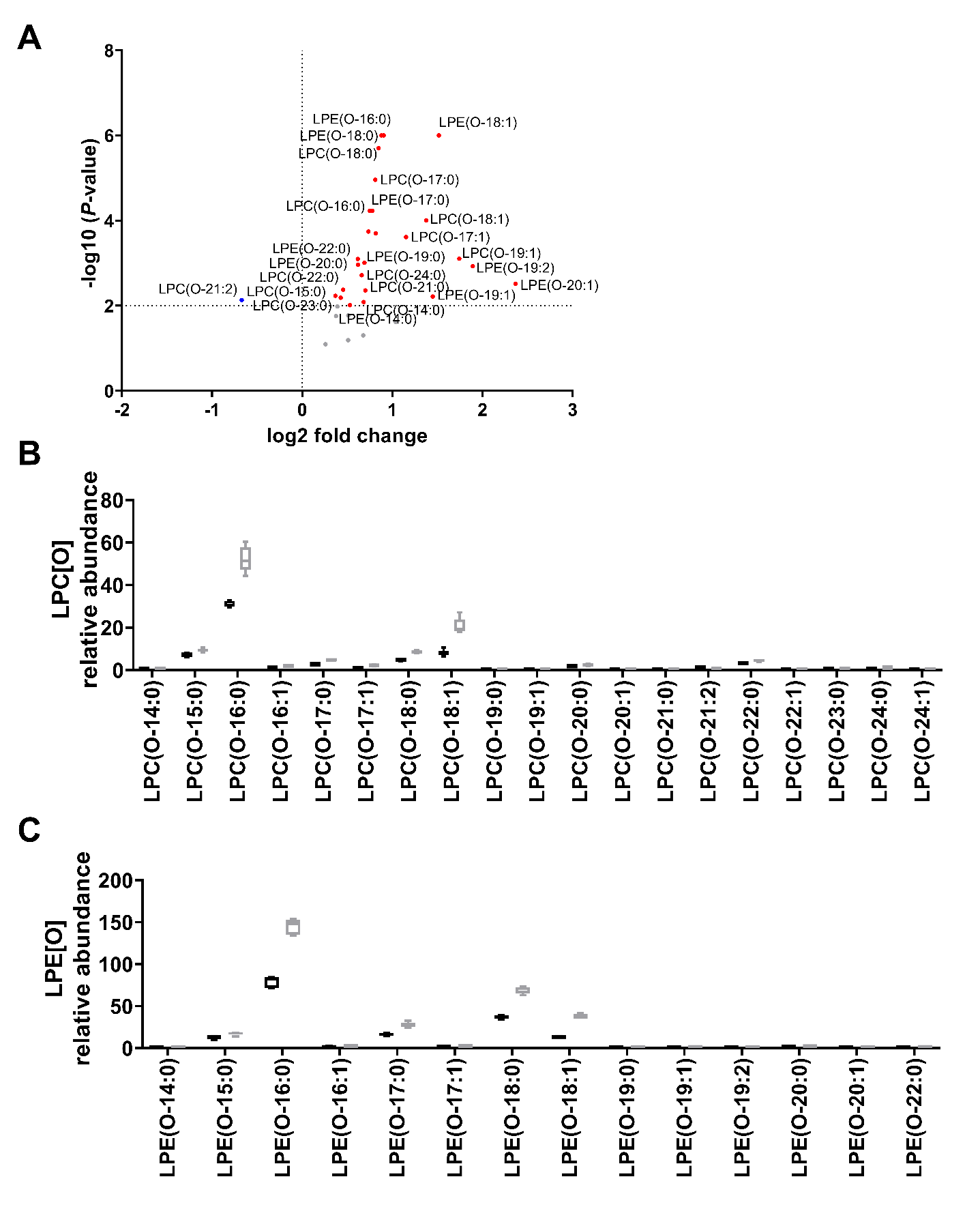


**Supplemental Fig. S8. Detailed analysis of carbon chain length and degree of desaturation of single LPC[O] and LPE[O] species upon *Agmo* knockdown in mature adipocytes.** **A)** Volcano plot depicting the lipidomics data of LPC[O] and LPE[O] at day 11 of adipocyte differentiation. Significance cut-oﬀ is *P* < 0.01 as indicated by the horizontal dotted line. The vertical dotted line separates the log2(fold change) of decreased (blue dots) and increased (red dots) lipid species. Boxplots showing the relative abundances of **B)** LPC[O] and **C)** LPE[O] at day 11 of adipocyte differentiation in the sh*Luc* and sh*Agmo* cell lines.
